# Mercaptophenylboronic Acid‐Mediated Nanozyme Immunochromatographic Assay for Simultaneous Detection of Respiratory Bacteria and Virus

**DOI:** 10.1002/advs.202502574

**Published:** 2025-05-08

**Authors:** Qing Yu, Jiaxuan Li, Shuai Zheng, Yajin Hu, Benshun Tian, Meirou Lu, Bing Gu, Chongwen Wang

**Affiliations:** ^1^ Department of Clinical Laboratory Medicine, Guangdong Provincial People’s Hospital (Guangdong Academy of Medical Sciences) Southern Medical University Guangzhou 510006 China; ^2^ School of Medicine South China University of Technology Guangzhou Guangdong 510000 China; ^3^ Hefei Institute of Physical Science Chinese Academy of Sciences Hefei 230036 China

**Keywords:** biomimetic magnetic nanozyme, 4‐mercaptophenylboronic acid, immunochromatographic assay, respiratory pathogens, universal detection

## Abstract

Rapid, sensitive, and accurate detection of respiratory pathogens is essential yet challenging. Here, this study presents a universal nanozyme immunochromatographic assay (ICA) that utilizes the broad binding capacity of 4‐mercaptophenylboronic acid (MPBA) for glycosylated molecules on bacterial and viral surfaces, along with the colorimetric‐catalytic dual enhancement capability of multi‐tentacle magnetic nanozyme (FeAu@AuIr), to achieve ultrasensitive, wide‐range, and simultaneous detection of multiple respiratory pathogens. The virus‐like biomimetic FeAu@AuIr with multi‐tentacle shell is prepared by sequentially assembling a layer of large Au nanoparticles and a layer of highly catalytic 5 nm AuIr nanoparticles on 160 nm Fe_3_O_4_ surface, greatly enhancing the universal capture/detection capability of detection system for both bacteria and virus. By simultaneously detecting two significant respiratory bacteria (*Streptococcus pneumoniae* and *Pseudomonas aeruginosa*) and one respiratory virus (Severe Acute Respiratory Syndrome Coronavirus 2), this study demonstrates that combination of FeAu@AuIr‐MPBA probe and antibody‐modified ICA strips can achieve ultrasensitive and highly specific detection of different respiratory pathogens, with sensitivity and detection range improved by more than 238 times compared to existing colorimetric ICA methods. Moreover, the practical utility of the FeAu@AuIr‐based ICA is validated through testing 170 positive clinical respiratory samples, underscoring its considerable potential for real‐time pathogen detection in both clinical and field settings.

## Graphical abstract

This study developes a universal colorimetric ICA platform that integrates broad‐spectrum recognition by 4‐mercaptophenylboronic acid with dual signal enhancement—both colorimetric and catalytic—mediated by a biomimetic magnetic nanozyme. This platform, for the first time, enables simple, convenient, ultrasensitive, and simultaneous screening of multiple respiratory bacteria and virus.

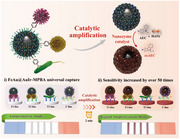

## 1 Introduction

Respiratory tract infections are prevalent diseases primarily caused by two major categories of microorganisms: bacteria and viruses.^[^ ^1^ ^]^ These infections exhibit high incidence rates, rapid transmission, and a propensity to develop into severe cases, thereby posing a significant threat to public health globally.^[^ ^2^ ^]^ For instance, *Streptococcus pneumoniae* (*S. pneumoniae*), a key bacterial pathogen, is responsible for ≈500 000 deaths annually, predominantly affecting children under five and the elderly.^[^ ^3^ ^]^ Since the identification of Severe Acute Respiratory Syndrome Coronavirus 2 (SARS‐CoV‐2) in late 2019, global infections have exceeded 700 million, resulting in over 7 million deaths.^[^ ^4^ ^]^ Although bacterial and viral respiratory infections share similar clinical manifestations—primarily coughing, difficulty breathing, and fever—their treatment strategies differ fundamentally. Misdiagnosis or delayed diagnosis can lead to severe consequences, including postponed treatment and a heightened risk of serious illness and mortality.^[^ ^5^ ^]^ Furthermore, misclassifying viral infections as bacterial may trigger antibiotic misuse, thereby exacerbating antimicrobial resistance.^[^ ^6^ ^]^ Hence, rapid and accurate pathogen diagnosis is crucial for preventing misdiagnosis and ultimately saving lives.

Current nucleic acid detection methods, such as real‐time fluorescence quantitative polymerase chain reaction (qPCR) and gene sequencing, represent the primary tools for clinical diagnosis of respiratory pathogens.^[^ ^7^ ^]^ However, these techniques rely on nucleic acid amplification and face multiple challenges in rapid diagnostics. In particular, they require contamination‐free environments, involve long processing times (3–24 h), demand highly skilled technicians, and depend on costly equipment (with PCR instruments priced above $5000 and sequencing systems exceeding $100 000).^[^ ^8^ ^]^ Moreover, the current gold standard, qPCR, cannot simultaneously detect bacteria and viruses due to inherent structural differences between these microorganisms. For instance, bacterial detection typically necessitates cell lysis and subsequent DNA extraction, whereas the diagnosis of most respiratory viruses—being RNA viruses—requires reverse transcription of viral RNA into DNA prior to amplification.^[^ ^9^ ^]^ Consequently, there is an urgent need for a rapid, accurate, and cost‐effective diagnostic technology that can concurrently differentiate between bacterial and viral infections.

Immunochromatographic assay (ICA) is a rapid immunodetection technique that leverages specific antigen–antibody reactions on a test strip. It offers rapid, simple, and cost‐effective detection of various biochemical targets and is widely applied in food safety, environmental monitoring, and home self‐testing.^[^ ^10^ ^]^ Theoretically, ICA holds considerable promise for rapid clinical diagnosis of respiratory infections based on several unique advantages: i) low cost (less than $2) enabling large‐scale screening; ii) robust tolerance that eliminates the need for contamination‐controlled facilities, thus allowing use in diverse on‐site settings; iii) simplicity and portability, independent of large instruments; and iv) direct detection of pathogen structural proteins or cells via antigen–antibody interactions, which facilitates simultaneous diagnosis of viruses and bacteria by incorporating multiple antibody‐modified test lines onto a single test strip. However, current ICA technology exhibits two major technical limitations in pathogen detection that preclude its full performance. First, traditional nanolabels—such as colloidal gold nanoparticles and latex beads—generate only moderate colorimetric signals and exhibit limited stability in complex matrices, resulting in unsatisfactory detection sensitivity.^[^ ^11^ ^]^ Although new optical signal materials, including fluorescent microspheres, quantum dots, and surface‐enhanced Raman scattering (SERS) tags, can improve sensitivity, these approaches require specialized instruments for signal reading, thus diminishing the inherent convenience of ICA.^[^ ^12^ ^]^ Second, owing to the large molecular mass of microorganisms, current ICA methods predominantly rely on a double‐antibody sandwich strategy for pathogen detection.^[^ ^13^ ^]^ In this approach, one antibody (capture antibody) labels the detection nanoprobes for specific target recognition, while another antibody (detection antibody) is used to modify the test line (T‐line) to immobilize the formed immune complexes. Screening distinct antibody pairs for different pathogens is labor‐intensive, time‐consuming, and costly. Moreover, antibodies conjugated on the nanoprobe surface are susceptible to the adverse effects of complex samples (such as high ionic concentrations and extreme pH conditions), leading to reduced activity and compromised stability and accuracy of the assay. To date, no ICA technique has been developed that can simultaneously detect both bacteria and viruses with high sensitivity and simplicity.

In recent years, nanozymes, a class of metal‐based nanomaterials exhibiting peroxidase‐like activity, have shown great application prospects in in vitro diagnostics and in vivo therapy due to their powerful catalytic ability, good biocompatibility, chemical stability, and excellent optoelectronic properties.^[^ ^14^ ^]^ Notably, nanozymes can act as multifunctional signal nanolabels, delivering superior colorimetric performance on test strips and enhancing the sensitivity of colorimetric immunochromatographic assays (ICA) by several tens of times through a simple one‐step catalytic reaction.^[^ ^15^ ^]^ Additionally, previous studies have demonstrated that phenylboronic acid and its derivatives [e.g., 4‐mercaptophenylboronic acid (MPBA)] can effectively detect bacteria through the binding between boronic acid ligands and the peptidoglycan in bacterial cell walls.^[^ ^16^ ^]^ Our recent research further illustrated that a phenylboronic acid‐modified magnetic quantum dot probe can broadly recognize viral envelope glycoproteins, facilitating high‐sensitivity on‐site detection of three emerging viruses using an ICA strip.^[^ ^17^ ^]^ Inspired by these findings, we have developed a universal colorimetric ICA platform that integrates broad‐spectrum recognition by MPBA with dual signal enhancement—both colorimetric and catalytic—mediated by a biomimetic magnetic nanozyme. This platform, for the first time, enables simple, convenient, ultrasensitive, and simultaneous screening of multiple respiratory bacteria and virus in complex clinical specimens. A virus‐like magnetic nanozyme (FeAu@AuIr) was designed and synthesized, featuring a 160 nm Fe_3_O_4_ core that provides rapid magnetic enrichment capability; a layer of 15 nm Au nanoparticles (Au_15_) that enhances the colorimetric performance of the nanolabel and offers a greater surface area for loading additional nanozyme particles; and a multi‐tentacle shell composed of 5 nm AuIr particles (AuIr_5_) that delivers high catalytic activity and provides ample binding sites for MPBA modification to further boost pathogen capture. We demonstrated that the MPBA‐modified FeAu@AuIr nanozyme can efficiently capture bacteria and virus within 5 min and enable dual‐mode colorimetric‐catalytic detection on antibody‐modified test strips with high sensitivity and specificity. We further validated the broad applicability and multiplex diagnostic potential of our approach by concurrently detecting two key respiratory bacteria, *S. pneumoniae* and *Pseudomonas aeruginosa (P. aeruginosa*), and the highly pathogenic respiratory virus SARS‐CoV‐2. Under catalytic conditions, the FeAu@AuIr‐MPBA mediated nanozyme ICA achieved detection sensitivities of 21 cells mL^−1^ for *S. pneumoniae*, 17 cells mL^−1^ for *P. aeruginosa*, and 1.2 pg mL^−1^ for SARS‐CoV‐2, representing ≈16–73 times improvement compared to pre‐catalysis results and a 238–416 times enhancement over traditional double‐antibody sandwich colorimetric ICA methods. Furthermore, the high accuracy and stability of the FeAu@AuIr‐based ICA platform were validated through testing actual clinical respiratory samples, demonstrating the broad potential of our method as a next‐generation, real‐time, and universal microbial detection technology.

## 2 Results and Discussion

### 2.1 Synthesis and Characterization of Multi‐Tentacle Magnetic Nanozyme

**Scheme 1:**
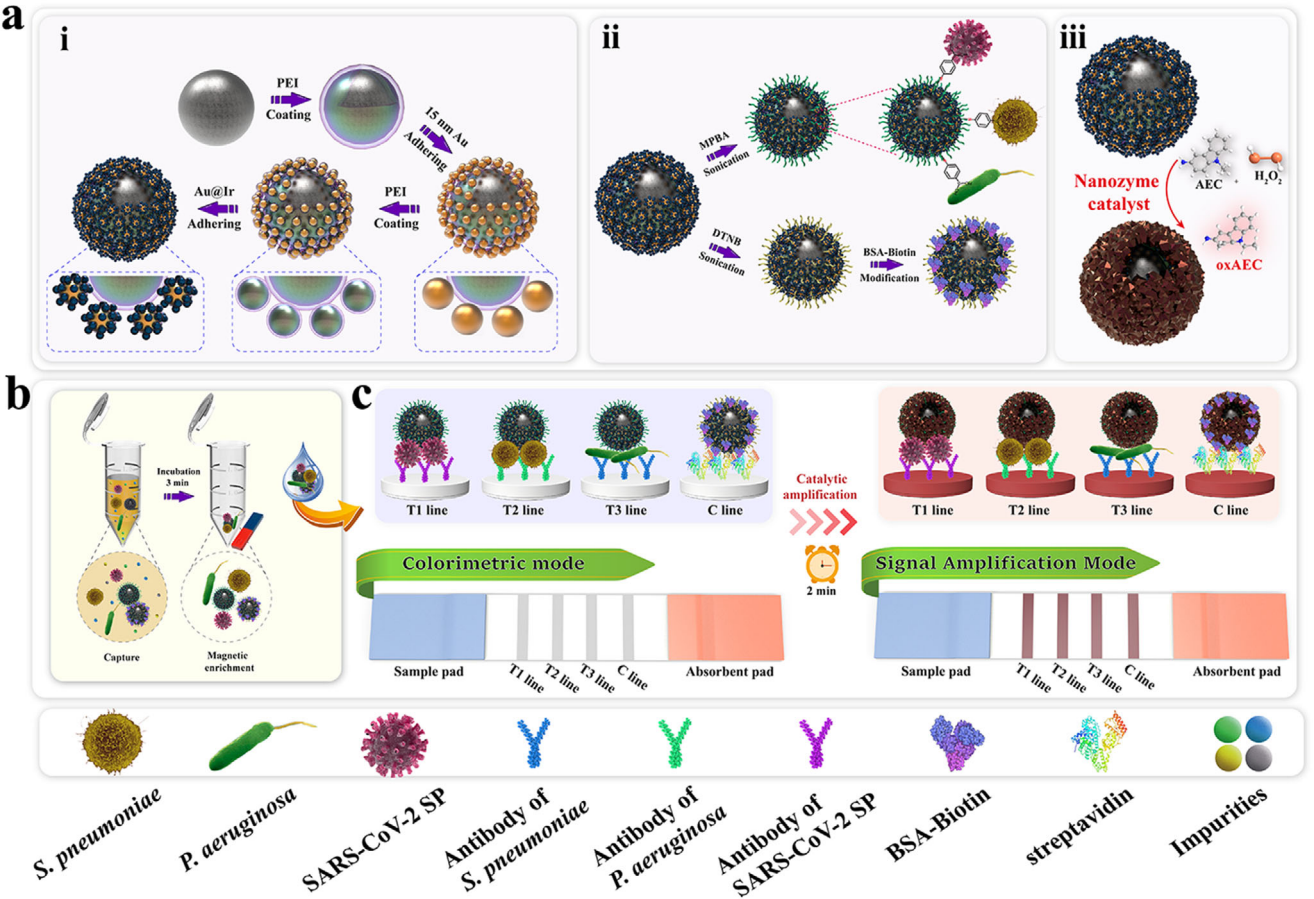
a) Schematic diagram of i) the preparation process for FeAu@AuIr multi‐tentacle magnetic nanozyme, ii) the process for modifying MPBA and BSA‐Biotin on FeAu@AuIr surface, and iii) the catalytic mechanism of the FeAu@AuIr nanozyme for AEC. Schematic illustration of b) FeAu@AuIr‐MPBA for respiratory bacteria/virus universal capture and c) FeAu@AuIr‐ICA for colorimetric‐catalytic dual‐mode detection of pathogens.

Herein, we designed and synthesized a multi‐tentacle magnetic nanozyme (FeAu@AuIr) with MPBA surface modification to enable on‐site, rapid, simultaneous, and ultrasensitive detection of both bacteria and virus using a colorimetric ICA platform. As depicted in **Scheme** **1**a(i), the FeAu@AuIr nanocomposite is fabricated by sequentially coating a dense layer of Au_15_ nanoparticles (NPs) followed by a layer of AuIr_5_ nanozyme onto monodisperse 160 nm Fe_3_O_4_ magnetic nanoparticles (MNPs) via cationic polymer polyethyleneimine (PEI)‐mediated electrostatic adsorption. This strategy endows the proposed multi‐tentacle nanozyme with strong magnetic responsiveness, an enlarged relative surface area, high‐density catalytic sites, and dual‐enhanced colorimetric and magnetic performance.

**Figure 1:**
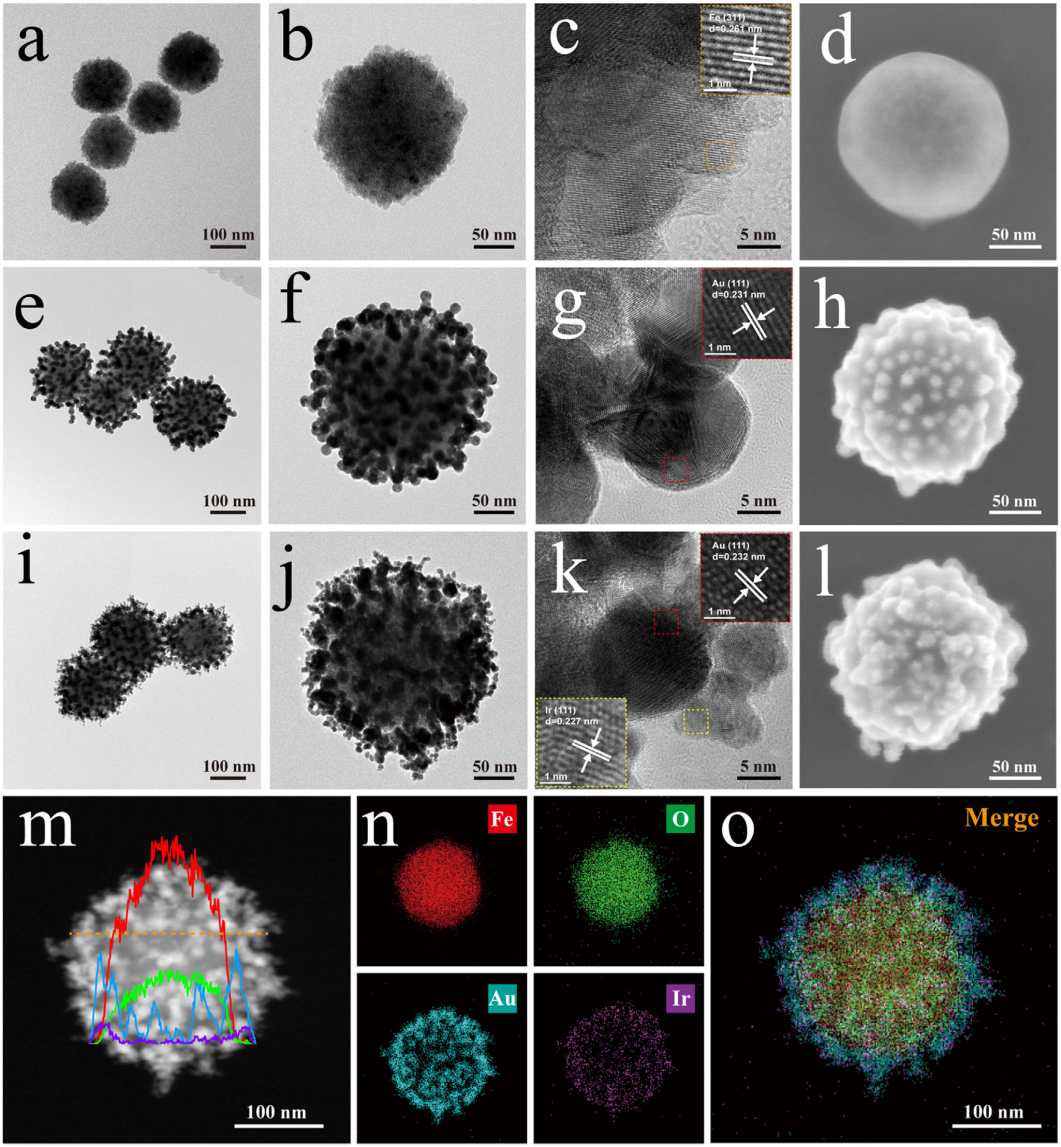
Morphological characterization of FeAu@AuIr multi‐tentacle magnetic nanozyme. Typical TEM images of a) Fe_3_O_4_ MNPs, e) FeAu MNPs, and i) FeAu@AuIr nanozymes, with their corresponding magnified TEM images of individual particle in (b,f,j), respectively. Locally magnified TEM images of the surface structure of c) Fe_3_O_4_, g) FeAu, and k) FeAu@AuIr nanocomposite. Representative SEM images of (d) Fe_3_O_4_, h) FeAu, and l) FeAu@AuIr nanozyme. m) EDS elemental line scan and n) elemental mapping results of individual multi‐tentacle FeAu@AuIr.

**Figure 2:**
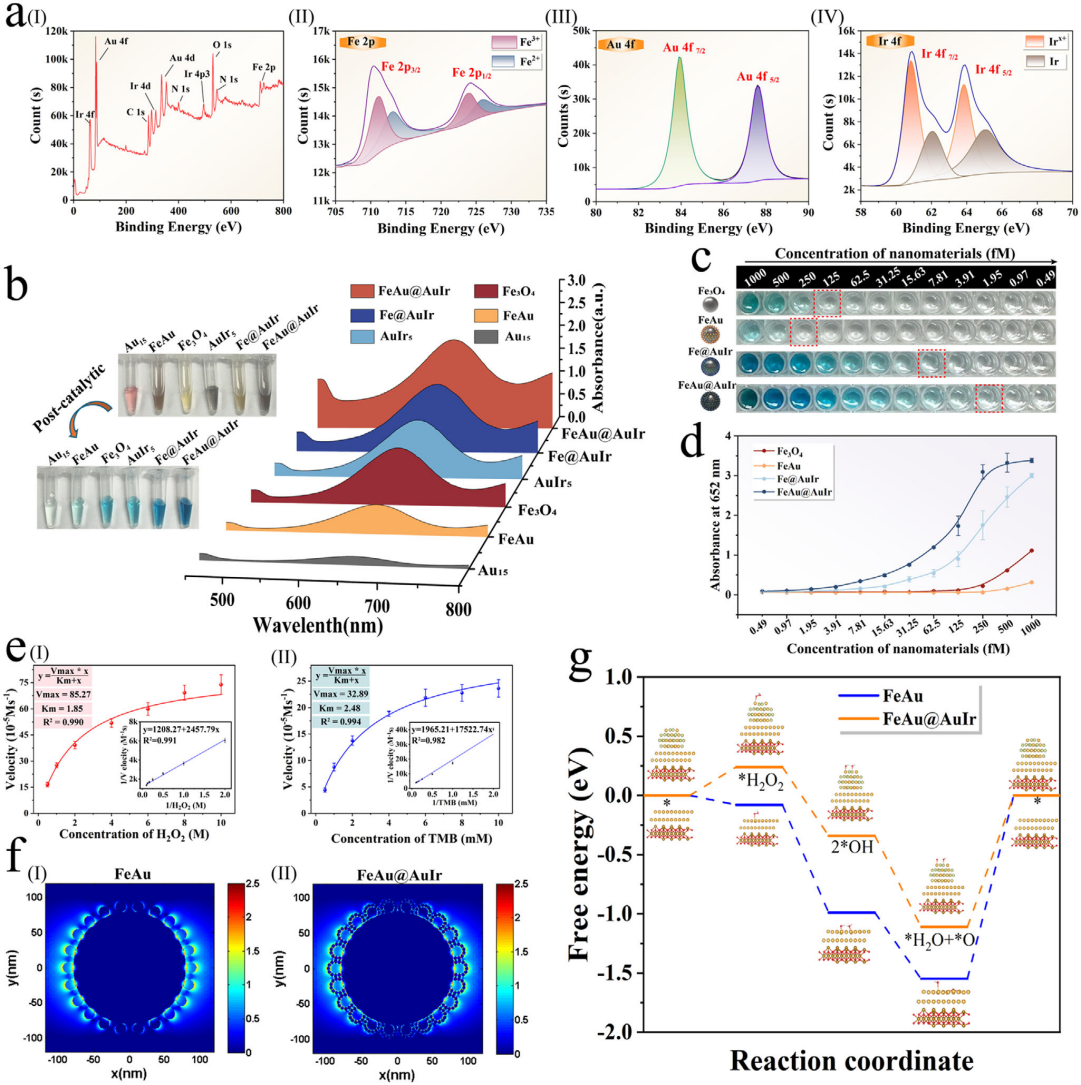
Characterization of the physical/chemical properties of multi‐tentacle FeAu@AuIr nanozyme. a) Wide−scan XPS spectra (I) and corresponding high−resolution Fe 2p (II), Au 4f (III), and Ir 4f (IV) spectra of FeAu@AuIr. b) Photograph and UV–vis absorption spectra of TMB catalyzed by different nanomaterials including Au_15_, FeAu, Fe_3_O_4_, AuIr_5_, Fe@AuIr, and multi‐tentacle FeAu@AuIr. c) Post‐catalytic photographs of the catalytic oxidation of TMB (1 mM) solution in the presence of H_2_O_2_ by Fe_3_O_4_, FeAu, Fe@AuIr, and FeAu@AuIr at 0.49−1000 fM and d) corresponding absorbance values at 652 nm after the catalytic oxidation. e) Steady‐state kinetic analysis of FeAu@AuIr: (I) plot of reaction velocity versus H_2_O_2_ concentration when TMB concentration is fixed, and (II) plot of reaction velocity versus TMB concentration when H_2_O_2_ concentration is fixed. Insets: Lineweaver–Burk plot. f) Calculated near‐field electromagnetic field distributions of (I) FeAu and (II) FeAu@AuIr nanozymes. g) Free energy diagrams of FeAu and FeAu@AuIr during catalysis.

The transmission electron microscopy (TEM) images in **Figure** **1**a,e,i reveal the typical morphologies of prepared Fe_3_O_4_, Au_15_‐coated Fe_3_O_4_ (FeAu), and FeAu@AuIr nanozymes, respectively. The Fe_3_O_4_ MNPs prepared by the co‐precipitation method exhibit a nearly standard spherical structure, with a uniform particle size distribution and an average size of 160 nm, as well as good dispersibility (Figure 1a). Benefiting from the strong electronegativity of Fe_3_O_4_ MNPs, the polyamine polymer PEI can rapidly self‐assemble on their surface under ultrasonic driving, resulting in a strong positive charge on the surface of the magnetic particles. The zeta potential results in Figure S1 (Supporting information) indicate that the surface charge of PEI‐coated Fe_3_O_4_ (Fe@PEI) changed from a negative potential (−36.48 mV) to a strong positive potential (26.57 mV), confirming the successful formation of the PEI shell. Previous works indicate that PEI‐modified nanomaterials can firmly adsorb a large number of negatively charged small particles (e.g., Au NPs, Pt NPs, Au@Pt, Au@Ag) through electrostatic interactions.^[^ ^18^ ^]^ In this study, we selected high electronegativity Au_15_ NPs as a multifunctional element to prepare multi‐tentacle nanozymes, which can provide powerful colorimetric activity and a large reaction interface for loading small catalytic particles (Figure S2, Supporting information). The prepared FeAu MNPs have a dense layer of Au NPs on their surface, exhibiting a typical core‐satellite structure (Figure 1e). The outermost AuIr_5_ nanozymes can be easily assembled onto the surface of FeAu MNPs through a process of repeated PEI assembly and small satellite adsorption, thereby transforming the core‐satellite bimetallic structure (FeAu) into a core‐satellite‐nanotentacle trimetallic structure (FeAu@AuIr). As shown in **Figure** **2**i, the resulting FeAu@AuIr nanozyme exhibit a virus‐like surface morphology, with numerous spike structures arranged in a regular pattern across the surface. TEM images (Figure 1b,f,j) and scanning electron microscope (SEM) images (Figure 1d,h,l) both reveal that with the continuous self‐assembly of Au_15_ and AuIr_5_ NPs, the surface of individual magnetic nanozyme becomes increasingly rough, and the multi‐tentacle structure becomes increasingly prominent. The surface details of Fe_3_O_4_, FeAu, and FeAu@AuIr are revealed in Figure 1c,j,k, respectively. These magnified TEM images clearly show that Au_15_ NPs are densely arranged on the surface of FeAu (Figure 1g), while multiple AuIr_5_ NPs are grafted onto each Au_15_ on the surface of FeAu@AuIr (Figure 1k). In addition, the Fe_3_O_4_ core, Au_15_ satellites, and AuIr_5_ NPs (Au core and Ir shell) of the multi‐tentacle magnetic nanozyme exhibit lattice spacings of 0.261, 0.231, 0.232, and 0.227 nm, corresponding to (fcc) Fe (311), Au (111), Au (111), and Ir (111), respectively.^[^ ^19^ ^]^ The energy‐dispersive spectroscopy (EDS) element line scanning results as well as the element mapping results for a typical FeAu@AuIr nanocomposite are presented in Figure 1m,n, respectively. These results clearly show the hierarchical distribution of Au (blue) and Ir (purple) elements on the surface of Fe (red) and O (green) elements in FeAu@AuIr nanostructure, revealing the spatial structure and main components of the proposed multi‐tentacle nanozyme. The above experimental results confirm the successful synthesis of a magnetic core‐Au satellites‐AuIr multi‐tentacled shell nanozyme.

Nanoparticle tracking analysis (NTA) result (Figure S3, supporting information) reveals that after the continuous coating of Au_15_ and AuIr_5_ NPs, the prepared FeAu@AuIr nanocomposites still exhibit excellent dispersibility, with particle size increasing from 160 to 200 nm. Moreover, due to the use of 160 nm Fe_3_O_4_ particles with strong magnetic responsiveness as carriers, the multi‐tentacle FeAu@AuIr nanozyme demonstrated excellent magnetic properties. As shown in Figure S4a (supporting information), after consecutive loading of Au_15_ and AuIr_5_, the FeAu@AuIr nanozyme still maintained a strong saturation magnetization (57.1 emu g^−1^). Under an external magnetic field, the prepared magnetic nanozyme could achieve complete enrichment within 1 min (Figure S4b, supporting information), proving that our designed biomimetic nanozyme can serve as an efficient tool for the rapid separation of respiratory pathogens.

Our biomimetic multi‐tentacle FeAu@AuIr nanozyme, designed based on virus particles, features numerous Au_15_‐AuIr_5_ spike structures on its surface as colorimetric‐catalytic dual‐enhancement sites, thus exhibiting excellent optical and physicochemical properties. The surface elemental composition of nanozymes directly determines their catalytic ability, so we first investigated the catalytic element distribution on the surface of FeAu@AuIr nanozymes using X‐ray photoelectron spectroscopy (XPS). In the wide scan XPS spectra [Figure 2a‐(I)] and high‐resolution XPS spectra [Figure 2a‐(II–IV)], strong signal peaks for Ir, Au, C, N, and O elements are observed, along with weak signal peaks for Fe, indicating that the outer layer of the multi‐tentacle magnetic nanozyme is composed of AuIr, Au, and PEI. Theoretically, a high concentration of Ir is beneficial enhancing the catalytic activity of nanozyme. Next, we evaluated the colorimetric and catalytic properties of FeAu@AuIr, as these are crucial for determining the detection performance of the nanozyme on an ICA platform. The solution colors of different nanomaterials [Au_15_, AuIr_5_, Fe_3_O_4_, FeAu, AuIr_5_‐coated Fe_3_O_4_ (Fe@AuIr)] and multi‐tentacle FeAu@AuIr are shown in the inset (above) of Figure 2b. The color of the FeAu solution is clearly deeper than that of Fe_3_O_4_ and Fe@AuIr, indicating that surface‐modified Au_15_ NPs can significantly enhance the colorimetric performance of Fe_3_O_4_, while encapsulating 5 nm AuIr NPs can only slightly improve the colorimetric capability of Fe_3_O_4_. Meanwhile, as Au_15_ and AuIr_5_ NPs were successively wrapped on the surface of Fe_3_O_4_, the absorbance of the magnetic nanozyme solution increased obviously (Figure S5a, Supporting information). The FeAu@AuIr nanozyme on the nitrocellulose (NC) membrane also shows a greatly higher colorimetric signal than Fe_3_O_4_, and a slightly higher colorimetric signal than FeAu (Figure S5b, Supporting information). These results indicate that the colorimetric capability of the proposed FeAu@AuIr is primarily derived from the assembled Au_15_ NPs, and is further enhanced through the grafting of AuIr_5_ nanozymes. Subsequently, we used 3,3′,5,5′‐ tetramethylbenzidine (TMB) as a chromogenic substrate to evaluate the peroxidase‐like activity of the magnetic nanozymes, as its oxidation product (oxTMB) produces a distinct UV–vis absorption peak at 652 nm, making it highly suitable for comparing catalytic activity of different nanomaterials. As shown in the inset (below) of Figure 2b, after the addition of TMB and H_2_O_2_, the solution colors of the Fe_3_O_4_, AuIr_5_, Fe@AuIr, and FeAu@AuIr groups changed from colorless to deep blue within 10 s, while the FeAu group solution exhibited a light blue color, and the solution color of the Au_15_ group remained almost unchanged. The solution color and UV–vis absorption peak at 652 nm indicate that the Fe@AuIr particles exhibit stronger catalytic ability for generating oxidized oxTMB compared to individual Fe_3_O_4_ and AuIr NPs, but are still weaker than FeAu@AuIr with a multi‐tentacled shell (Figure 2b). This phenomenon demonstrates that although Au_15_ does not exhibit obvious catalytic activity, it can provide a larger surface area to load more AuIr_5_ NPs, thereby generating more active catalytic sites. In addition, the FeAu@AuIr nanozymes maintain good catalytic activity and structural stability in aqueous solutions with pH ranging from 3 to 13, indicating that they can operate stably under various environmental conditions (Figure S6, Supporting information). We further investigated the catalytic specificity of the magnetic nanozymes for substrates. Results showed that the catalytic enhancement experiments based on FeAu@AuIr nanozyme require both TMB and H_2_O_2_ as substrates (Figure S7, Supporting information).

To determine the minimum catalytic concentration of the four different magnetic nanocomposites (Fe_3_O_4_, FeAu, Fe@AuIr, and FeAu@AuIr), we reacted magnetic enzymes at various concentrations (1000–0.49 fM) with the TMB/H_2_O_2_ mixed solution. The calculation method for determining magnetic nanozyme concentration is described in Section S1.8 (Supporting information). As the concentration of the four magnetic nanozymes decreases, the solution color gradually changes from blue to colorless (Figure 2c), while the absorbance of the reaction solution at 652 nm correspondingly decreases (Figure 2d). The visual detection limits for TMB catalyzed by Fe_3_O_4_, FeAu, Fe@AuIr, and FeAu@AuIr are 125, 250, 7.81, and 1.95 fM, respectively, by observing the blue signal with the naked eye. In addition, steady‐state kinetic experiments are used to quantify the peroxidase‐like activity and substrate binding affinity of nanozymes.^[^ ^20^ ^]^ In this method, the Michaelis constant (Km) represents the affinity for substrate binding, while the maximum reaction rate (Vmax) indicates catalytic activity. As shown in Figure 2e, the V_max_ and K_m_ of FeAu@AuIr for H_2_O_2_ are 85.27 M s^−1^ (I) and 1.85 mm, respectively, while the V_max_ and K_m_ of FeAu@AuIr for TMB are 32.89 M s^−1^ and 2.48 mm (II), respectively. Compared to the enzyme activity assessment results of Fe_3_O_4_, FeAu, and Fe@AuIr nanomaterials (Figure S8, Supporting information), the FeAu@AuIr nanocomposite exhibits the highest V_max_ value and the lowest K_m_ value for the H_2_O_2_/TMB substrates, further demonstrating the highest biocatalytic activity of proposed multi‐tentacle magnetic nanozyme. We further revealed the mechanism of catalytic activity enhancement in the FeAu@AuIr nanozyme from a theoretical perspective. We first employed the finite‐difference time‐domain (FDTD) simulation to study the spatial distribution of surface electric fields for FeAu and FeAu@AuIr nanocomposites (Section S1.10, Supporting Information). The results showed that after grafting AuIr_5_ NPs onto the surface of FeAu, additional and stronger localized electromagnetic (EM) fields are generated on the multi‐tentacled shell. The maximum EM enhancement factors for FeAu and FeAu@AuIr are 284‐fold and 340‐fold, respectively, confirming that the introduction of AuIr_5_ NPs enhanced the intrinsic electron donor properties of the magnetic nanozyme. Notably, previous studies have shown that improving the electron donor properties of nanostructures can provide higher energy for catalytic reactions, thereby effectively enhancing the catalytic activity of nanozymes.^[^ ^21^ ^]^ Therefore, the adsorption of small AuIr_5_ nanozymes onto FeAu surface not only increases the number of catalytic sites, but also enhances the catalytic reaction rate by optimizing electronic performance. We roughly calculated the number of AuIr NPs loaded on the surface of the multi‐tentacle FeAu@AuIr structure and confirmed that the introduction of an Au_15_ interlayer increases the quantity of AuIr NPs on the surface of the magnetic nanozyme to more than six times that of conventional spherical Fe@AuIr MNPs (Section S2 and Figure S9, Supporting information). This further demonstrates that the higher catalytic activity of FeAu@AuIr is attributed to the larger number of catalytic particles present on its surface.

We further employed density functional theory (DFT) calculation to verify the above conclusion. The construction details of DFT model are shown in Section S1.9 (Supporting Information), which are used to elucidate the effect of nanozyme surfaces on H_2_O_2_ decomposition efficiency. It is worth noting that steps 3 in the H_2_O_2_ catalytic process, namely the desorption of *OH/*O, are crucial for substrate oxidation and are considered the rate‐determining steps of catalysis.^[^ ^22^ ^]^ As shown in Figure 2g, the FeAu@AuIr nanocomposite exhibits obviously lower energy barriers in these steps compared to FeAu MNP, indicating that the generation of *OH radicals on the FeAu@AuIr surface faces less hindrance than on the FeAu particle surface. This result further demonstrates that AuIr_5_ grafting significantly enhanced the catalytic activity of magnetic nanozyme. Therefore, both experimental and theoretical results confirm that biomimetic nanozyme with a magnetic core‐Au satellites‐AuIr multi‐tentacled shell structure can simultaneously enhance the colorimetric and catalytic performance of traditional spherical magnetic nanozymes. This innovative design provides a new direction for the performance improvement and practical application of nanozymes.

The size of magnetic nanozymes has a significant impact on their performance, including magnetic properties, catalytic activity, and fluidity. Therefore, we systematically evaluated the detection performance of different magnetic nanozymes prepared using Fe_3_O_4_ cores of varying sizes (120–220 nm). The results showed that the FeAu@AuIr nanozyme, prepared with a 160 nm Fe_3_O_4_ core, is the optimal nanostructure for use in a nanozyme‐ICA platform for pathogen detection. (details are provided in Section S3 and Figure S10, Supporting Information).

### 2.2 Evaluation of Universal Capture/Detection Capability of MPBA‐Modified FeAu@AuIr Nanozyme for Respiratory Bacteria and Virus

**Figure 3:**
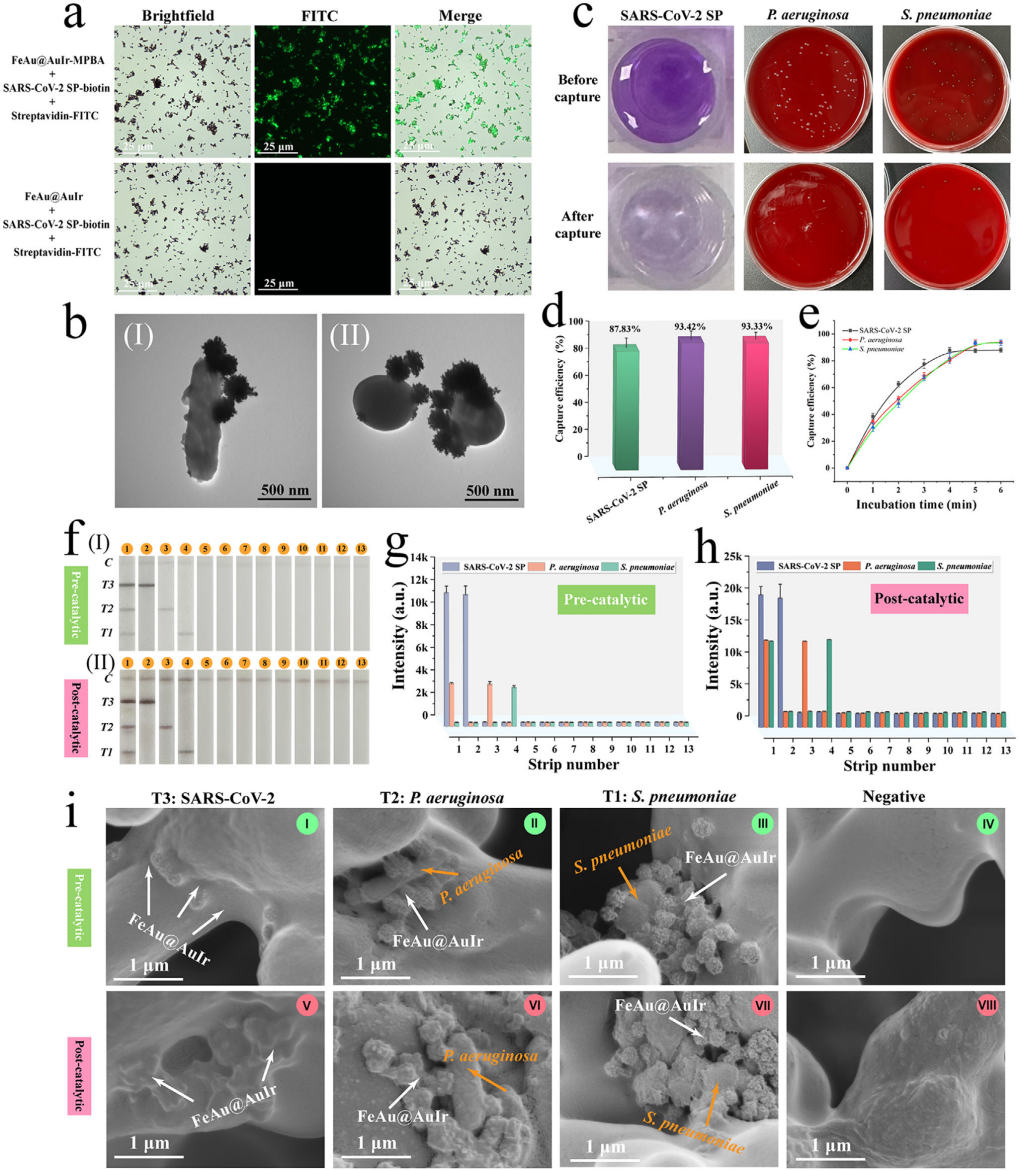
a) Fluorescence microscopy analysis for verification of the binding ability of FeAu@AuIr‐MPBA to SARS‐CoV‐2 SP. b) TEM images of *P. aeruginosa* (I) and *S. pneumoniae* (II) captured by FeAu@AuIr‐MPBA. c) BCA assay and plate culture results showing the concentration of remaining viral protein and bacteria in the supernatant before/after FeAu@AuIr‐MPBA capture, respectively. d) Calculated capture efficiency of FeAu@AuIr‐MPBA for three target pathogens. e) Capture efficiency of FeAu@AuIr‐MPBA versus incubation time. f) Photographs and g,h) measured colorimetric signals on the T‐lines of corresponding test strip before (I) and after (II) catalytic activation for evaluating the specificity of FeAu@AuIr‐ICA for different pathogens: 1) mixture of SARS‐CoV‐2 SP/*P. aeruginosa*/*S. pneumoniae*, 2) SARS‐CoV‐2 SP, 3) *P. aeruginosa*, 4) *S. pneumoniae*, 5) Flu A, 6) Flu B, 7) RSV, 8) HAdV, 9) *S. aureus*, 10) *M. pneumoniae*, 11) *K. pneumoniae* 12) *L. pneumophilia*, and 13) blank control. i) Typical SEM images of the T‐line internal area for SARS‐CoV‐2, *P. aeruginosa*, *S. pneumoniae*, and negative samples. (I–IV) and (V–VIII) are the images of test strips before and after catalysis, respectively.

MPBA can effectively bind to peptidoglycan in bacterial cell walls, enabling broad‐spectrum recognition of bacteria.^[^ ^23^ ^]^ Additionally, phenylboronic acid ligands have been found to broadly detect viruses by effectively binding to glycoproteins (such as spike proteins) on viral surfaces.^[^ ^17^ ^]^ Therefore, theoretically speaking, MPBA‐modified magnetic nanozymes have the ability to efficiently capture both bacteria and viruses simultaneously. To verify this hypothesis, we selected two common respiratory pathogens (*P. aeruginosa* and *S. pneumoniae*) and the spike protein (SP) of SARS‐CoV‐2 to test the universal capture capability of FeAu@AuIr‐MPBA for bacteria and virus. Notably, MPBA molecules can form stable Ir‐S bonds with the AuIr_5_ shell through their terminal thiol groups, thereby stably modifying the surface of the FeAu@AuIr nanozyme. The characteristic SERS spectrum of MPBA‐modified FeAu@AuIr material is shown in Figure S11 (Supporting information), where the main characteristic peaks of MPBA (1075 and 1584 cm^−1^) are clearly observed, confirming the successful modification with MPBA.^[^ ^24^ ^]^ First, the binding capability of FeAu@AuIr‐MPBA to viral glycoproteins and bacteria was verified using fluorescence microscopy and TEM techniques, respectively, as bacteria are suitable for TEM observation while proteins are too small to be observed directly. The FeAu@AuIr‐MPBA and bare FeAu@AuIr were incubated with biotin‐conjugated SARS‐CoV‐2 SP for 5 min, followed by magnetic separation and washing, then further reacted with streptavidin‐FITC fluorescent probe. **Figure** **3**a reveals that numerous green fluorescent molecules (streptavidin‐FITC) specifically bound to FeAu@AuIr‐MPBA after reacting with SARS‐CoV‐2 SP‐biotin, while no significant green fluorescence signals appeared on the unmodified FeAu@AuIr surface under the same conditions. These findings indicate that only MPBA‐modified FeAu@AuIr has the ability to capture the target viral protein. Meanwhile, TEM observations (Figure 3b) showed that MPBA‐modified FeAu@AuIr could rapidly bind to the surfaces of *P. aeruginosa* (I) and *S. pneumoniae* (II), demonstrating its ability to simultaneously recognize both Gram‐positive and Gram‐negative bacteria. Next, the capture efficiency of FeAu@AuIr‐MPBA for viral protein and two target bacteria was determined using the classical bicinchoninic acid (BCA) protein quantification method and plate counting method, respectively (Specific experimental details are provided in Section S1.11, Supporting Information). Based on the results of the BCA assay kit and plate counting method shown in Figure 3c, FeAu@AuIr‐MPBA for SARS‐CoV‐2 SP, *P. aeruginosa*, and *S. pneumoniae* achieved capture efficiencies of 87.83%, 93.42%, and 93.33%, respectively (Figure 3d). In addition, the high‐efficiency enrichment of bacteria/virus by FeAu@AuIr‐MPBA shows a clear time dependence, and an incubation time of 5 min is sufficient to achieve saturated capture of viral glycoproteins and bacteria (Figure 3e). These results strongly demonstrate that MPBA‐modified magnetic nanozyme possesses a high efficiency in capturing virus and bacteria, laying the foundation for the development of a universal detection platform for respiratory pathogens.

Given the excellent colorimetric/catalytic performance as well as broad‐spectrum pathogen capture capability of FeAu@AuIr‐MPBA, we further tested its feasibility for simultaneous detection of bacteria and virus on ICA platform. We first screened suitable catalytic substrates for FeAu@AuIr nanozyme‐based ICA by testing two most commonly used peroxidase chromogenic substrates, including TMB and AEC (3‐amino‐9‐ethylcarbazole). As shown in Figure S12 (Supporting information), the AEC substrate exhibits a clearer red signal on the test strip with a better signal‐to‐noise ratio (SNR). Scheme 1a‐(iii) illustrates the principle of FeAu@AuIr catalyzing AEC: when the multi‐tentacle nanozyme comes into contact with H_2_O_2_, it can rapidly decompose H_2_O_2_ into highly reactive hydroxyl radicals (*OH), which can further catalyze the oxidation of AEC, resulting in the formation of an insoluble reddish‐brown compound (oxAEC). The formed oxAEC is deposited abundantly around the detection zone, significantly enhancing the colorimetric signal intensity of the testing line (T‐line), thereby greatly improving the sensitivity of the colorimetric ICA technology. In addition, we found that the MPBA modification of FeAu@AuIr did not significantly influence the catalytic activity of the nanozyme, thereby facilitating the subsequent construction of nanozyme‐ICA (Figure S13, Supporting information).

Next, we designed a three‐channel ICA strip by immobilizing mouse monoclonal antibodies (mAbs) against *S. pneumoniae*, *P. aeruginosa*, and SARS‐CoV‐2 on three T‐lines on a NC membrane for the specific immunological detection of two target bacteria and one target virus (Scheme 1c). Additionally, we constructed a streptavidin‐modified control line (C‐line) on the NC membrane and used an additional bovine serum albumin (BSA)‐biotin modified FeAu@AuIr probe to accurately monitor the activity of three mAbs on the test strip, ensuring the effectiveness of the detection system [Scheme 1a‐(ii),c]. The detection process of universal ICA based on multi‐tentacle FeAu@AuIr is shown in Scheme 1b‐c: First, FeAu@AuIr‐MPBA is added to the sample solution for rapid and universal enrichment of bacteria and virus; second, the magnetically enriched FeAu@AuIr complexes are resuspended in the loading buffer and dropped onto the sample pad of the test strip for colorimetric‐catalytic dual‐mode chromatographic detection. When target virus or bacteria are present in the sample solution, they are effectively enriched by FeAu@AuIr‐MPBA, forming magnetic nanozyme‐pathogen complexes. These complexes are then specifically captured by mAbs pre‐modified in different T‐lines during chromatography, generating sandwich complexes at the specific detection zones. If no target microorganisms are present in the sample, the FeAu@AuIr‐MPBA probes will pass through all three T lines and ultimately be collected by the absorbent pad. Under normal conditions, FeAu@AuIr‐biotin, serving as a quality control probe, will be captured by streptavidin on the C‐line, producing stable signal to verify the validity of the experiment. After directly observing the colorimetric signals on the test strip, catalytic substrate solution can be added for signal amplification on the T‐lines, significantly improving sensitivity and detection range within a short time (within 2 min). Additionally, the colorimetric intensity produced on the T lines of nanozyme‐ICA can be rapidly quantified using ImageJ (Scheme 1c).

The selectivity and specificity of the constructed multi‐channel ICA were validated by detecting different target bacterial/viral samples and non‐target pathogens. Figure 3f‐(I,II) show the detection results of the nanozyme test strips before and after AEC catalytic amplification for mixed samples of *S. pneumoniae*/*P. aeruginosa*/SARS‐CoV‐2 (strip 1), SARS‐CoV‐2 sample (strip 2), *P. aeruginosa* sample (strip 3), *S. pneumoniae* sample (strip 4), as well as non‐target pathogen samples including influenza A virus (strip 5), influenza B virus (strip 6), respiratory syncytial virus (strip 7), adenovirus (strip 8), *Staphylococcus aureus* (strip 9), *Mycoplasma pneumoniae* (strip 10), *Klebsiella pneumoniae* (strip 11), and *Legionella pneumophilia* (strip 12), along with a blank control group (strip 13). We found that the corresponding colorimetric signals of the T lines on the FeAu@AuIr‐ICA test strips for positive samples were effectively enhanced (changing from gray to deep red) through chemical catalysis. Meanwhile, the nanozyme‐based test strips for all negative samples showed no visible T‐lines before and after catalysis. Figure 3g,h shows the colorimetric signal intensities measured on the T‐lines before and after catalysis using ImageJ software. No cross‐reactivity could be found between the FeAu@AuIr‐MPBA probe and three detection channels in the ICA platform. The results demonstrate that only samples containing target bacteria/virus could generate detectable signals on the corresponding detection zone of the FeAu@AuIr‐ICA strips. Moreover, all three test lines exhibited good selectivity for their target pathogens and excellent specificity against all non‐target pathogens. We further observed the internal structure of the positive and negative T‐line regions using SEM (Figure 3i). In the T‐line region used to detect specific virus and bacteria, we observed the FeAu@AuIr probe complex (I), the FeAu@AuIr‐*P. aeruginosa* complex (II), and the FeAu@AuIr‐*S. pneumoniae* complex (III), while no multi‐tentacle nanozyme NPs (IV) were found in the negative control group. Additionally, the SEM images of the corresponding T lines after catalytic AEC treatment showed a large amount of irregular precipitates surrounding the FeAu@AuIr nanozymes (IV‐VII), whereas no precipitate deposition was observed in the same region of the negative samples (VIII). These results clearly demonstrate that the black signal on the T line before catalysis is generated by specific antibodies capturing FeAu@AuIr complexes, while the enhanced colorimetric signal originates from the accumulation of insoluble oxAEC produced through catalysis by multi‐tentacle nanozymes.

To achieve optimal detection performance, we further optimized key experimental parameters of FeAu@AuIr‐ICA, including chromatography time (Figure S14a, Supporting information), substrate catalytic time (Figure S14b, Supporting information), antibody modification concentration on the T line (Figure S14c, Supporting information), catalytic system (Figure S14d, Supporting information), dose of tags (Figure S15a, Supporting information) running buffer composition (Figure S15b, Supporting information), and incubation time (Figure S15c, Supporting information). In short, the optimal experimental parameters for the established nanozyme‐ICA were confirmed as follows: i) 13 min of chromatography time and 2 min of catalysis time; ii) T‐line antibody concentrations of 1 and 1.2 mg mL^−1^ for virus and bacteria detection, respectively; iii) optimal substrate combination of H_2_O_2_/AEC = 4/6; iv) 4 µL of FeAu@AuIr‐MPBA probe (1 mg mL^−1^); v) 10 mm PBS containing 1% Tween 20 and 5% milk as the running buffer, and vi) 5 min of pathogen capture time. Based on these optimization experiments, the established universal ICA technology can achieve flexible, ultrasensitive, and simultaneous detection of bacteria/virus within 20 min through a colorimetric‐catalytic dual‐mode strategy mediated by FeAu@AuIr nanozymes.

### 2.3 Performance Evaluation of the Universal Nanozyme‐ICA for Simultaneous Detection of Bacteria and Virus

**Figure 4:**
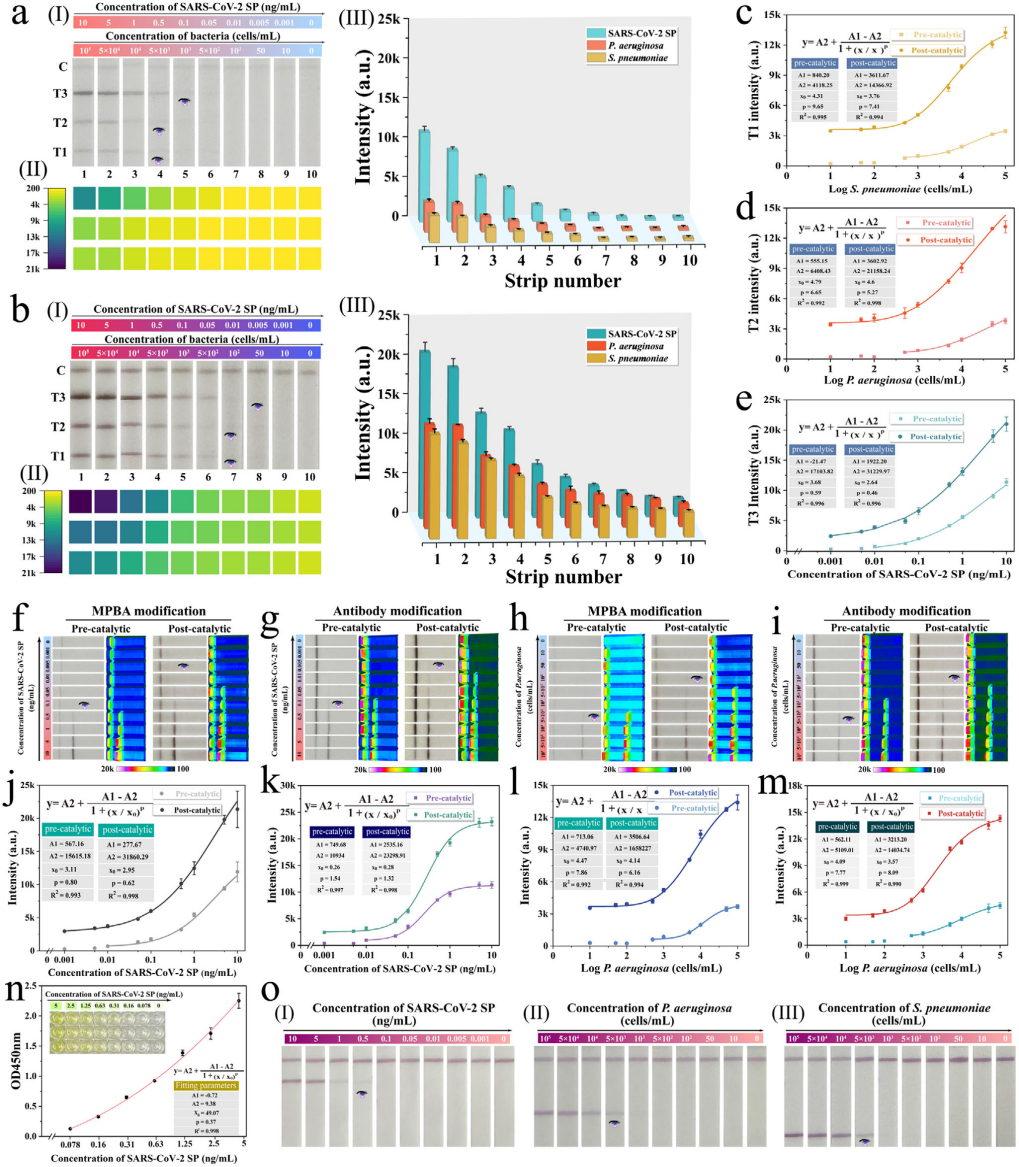
Evaluation of the simultaneous detection performance of the FeAu@AuIr‐ICA for three pathogens. Photographs (I), heatmaps (II), and detailed colorimetric intensities (III) on the T‐lines of the FeAu@AuIr‐ICA for simultaneous detection of *S. pneumoniae*, *P. aeruginosa*, SARS‐CoV‐2 SP, and before (a) and after (b) catalytic activation. Calibration curves of FeAu@AuIr‐ICA for *S. pneumoniae* (c), *P. aeruginosa* (d), and SARS‐CoV‐2 SP (e) before and after catalysis. Comparison of detection sensitivity between MPBA broad‐spectrum recognition strategy and dual‐antibody sandwich strategy in nanozyme‐ICA: Results of SARS‐CoV‐2 detection using FeAu@AuIr‐MPBA probe (f) and FeAu@AuIr‐antibody probe (g), and results of *P. aeruginosa* detection using FeAu@AuIr‐MPBA probe (h) and FeAu@AuIr‐antibody probe (i), and their corresponding fitting curves (j,k,l,m), respectively. n) ELISA result for SARS‐CoV‐2 SP. o) Conventional AuNP‐based ICA strips for SARS‐CoV‐2 SP (I), *P. aeruginosa* (II), and *S. pneumoniae* (III).

The main analytical performance (including sensitivity, detection range, and reproducibility) of the established magnetic nanozyme‐ICA for simultaneous determination of respiratory bacteria/virus was further evaluated. **Figure** **4**a‐(I,II) represents the photographs and colorimetric signal heatmaps of the test strips after directly detecting a series of mixed sample solutions containing different concentrations of *S. pneumoniae* (10^5^–10 cells mL^−1^), *P. aeruginosa* (10^5^–10 cells mL^−1^), and SARS‐CoV‐2 SP (10–0.001 ng mL^−1^). The colorimetric signals on all three T‐lines (T1/T2/T3) gradually decreased with decreasing concentrations of *S. pneumoniae*, *P. aeruginosa*, and SARS‐CoV‐2 SP. The black T lines remained visible to the naked eye even at low pathogen concentrations of 5 × 10^3^ cells mL^−1^ (*S. pneumoniae*)/5 × 10^3^ cells mL^−1^ (*P. aeruginosa*)/0.1 ng mL^−1^ (SARS‐CoV‐2 SP). The photos of test strips and T‐line signal intensities after the catalytic substrate enhancement are shown in Figure 4b‐(I,II). It can be seen that the colorimetric signals heatmaps in the T line area of the test strips were obviously increased, and the detection range for low concentrations of bacteria/virus was notably expanded. After AEC/H_2_O_2_ catalysis, the visual detection limits for *S. pneumoniae*, *P. aeruginosa*, and SARS‐CoV‐2 SP, as determined by T‐line colorimetric signals, were reduced to 100 cells mL^−1^, 100 cells mL^−1^, and 0.005 ng mL^−1^, respectively. These findings indicate that the test strips based on multi‐tentacle nanozyme showed a 50‐fold and 20‐fold increase in visual sensitivity for detecting bacteria and virus, respectively, before and after catalysis. Subsequently, we used ImageJ software to directly extract the specific colorimetric signals of the T‐line regions from photographs of the ICA strips before and after catalysis [Figure 4a‐(III),b‐(III)]. The results indicate that the FeAu@AuIr nanozyme can enhance the colorimetric signal of the T line by more than double through in situ catalysis using AEC/H_2_O_2_, and the catalytically enhanced colorimetric signal still maintains a positive correlation with the pathogen concentration. The corresponding sigmoidal calibration curves for two target respiratory bacteria and SARS‐CoV‐2 under direct/catalytic dual modes are shown in Figure 4c,d,e, respectively, which were established by plotting the colorimetric signals on each T‐line against target concentrations. Before catalysis, the limits of detection (LOD) of FeAu@AuIr‐mediated ICA for *S. pneumoniae*, *P. aeruginosa*, and SARS‐CoV‐2 SP are 993 cells mL^−1^, 1257 cells mL^−1^, and 0.020 ng mL^−1^, respectively, calculated based on the standard IUPAC definition (LOD = blank signal + 3 × standard deviation of blank measurements).^[^ ^25^ ^]^ After catalysis, the LODs of the test strips for *S. pneumoniae*, *P. aeruginosa*, and SARS‐CoV‐2 were reduced to 21 cells mL^−1^, 17 cells mL^−1^, and 0.0012 ng mL^−1^, respectively. Additionally, under the catalytic enhancement mode, the FeAu@AuIr‐ICA achieved a dynamic detection range of four orders of magnitude for all three target pathogens, with correlation coefficients (R^2^) exceeding 0.994, 0.998, and 0.996 for *S. pneumoniae*, *P. aeruginosa*, and SARS‐CoV‐2, respectively. These results demonstrate that combining ImageJ with our nanozyme‐ICA not only enables quantitative analysis but also improves the detection sensitivity by at least 47‐fold for bacteria and 16‐fold for virus compared to visual inspection.

Meanwhile, the proposed nanozyme‐ICA demonstrated excellent reproducibility. Figure S16 (Supporting information) shows the detection results of 5 independent groups of mixed samples at different concentrations of *S. pneumoniae*/*P. aeruginosa*/SARS‐CoV‐2 using FeAu@AuIr‐ICA test strips. As shown, the nanozyme‐strips exhibited consistent colorimetric/catalytic signals at each T‐line before and after catalysis, with a coefficient of variation ≤7.5%. The excellent reproducibility demonstrates the superiority of magnetic nanozyme‐mediated capture‐detection approach, which can eliminate interference from impurities in samples and improve the reliability of detection system.

In this study, MPBA replaces traditional antibodies as a universal recognition molecule for detecting bacteria and virus. Therefore, we need to evaluate the impact of the broad‐spectrum molecule (MPBA)‐based approach compared to the traditional double‐antibody sandwich approach on the performance of the FeAu@AuIr‐ICA platform for pathogen diagnosis. We used SARS‐CoV‐2 and *P. aeruginosa* as models to evaluate the performance of two methods for detecting virus and bacteria. Two immuno‐FeAu@AuIr labels were prepared by conjugating anti‐SARS‐CoV‐2 SP antibody and anti‐*P. aeruginosa* antibody to the FeAu@AuIr surface, respectively. The specific preparation method is described in Section S1.5 (Supporting Information). The usage of these immuno‐FeAu@AuIr probes in nanozyme‐ICA is consistent with that of FeAu@AuIr‐MPBA. Figure 4f,g show the direct (left)/catalytic (right) detection results of the same series of SARS‐CoV‐2 SP (10–0.001 ng mL^−1^) using ICA test strips based on FeAu@AuIr‐MPBA and immuno‐FeAu@AuIr nanozymes, while Figure 4h,i show the direct (left)/catalytic (right) detection results of the same series of *P. aeruginosa* (10^5^–10 cells mL^−1^) using nanozyme‐ICA based on FeAu@AuIr‐MPBA and immuno‐FeAu@AuIr. Obviously, in two parallel control experiments, the colorimetric/catalytic signals of the T‐line on test strips based on FeAu@AuIr‐MPBA probes showed similar trends to those with FeAu@AuIr‐antibody probes. Based on the heat maps of colorimetric signals obtained from test strips (Figure 4f–i) and corresponding fitted standard curves (Figure 4j–m), the LODs of the nanozyme‐ICA based on double‐antibody sandwich strategy for SARS‐CoV‐2 SP and *P. aeruginosa* were 16 pg mL^−1^ and 898 cells mL^−1^ before catalysis, and 0.8 pg mL^−1^ and 18 cells mL^−1^ after catalysis, respectively. Based on the above results, the MPBA‐modified FeAu@AuIr nanozyme demonstrates high specificity and accuracy in detecting target bacteria and virus, with detection sensitivity nearly equivalent to that of traditional antibody‐modified immunoprobes. In addition, the detection performance of the FeAu@AuIr‐ICA system remained stable for 90 days, which validates the excellent long‐term stability of our method (Figure S17, Supporting information). Further considering the broad‐spectrum recognition capability, low cost, and high stability of MPBA molecule, the multi‐tentacle nanozyme modified by MPBA undoubtedly offers performance advantages over traditional immunoprobes in the universal ICA system for pathogen detection.

In addition, we compared the performance of FeAu@AuIr‐ICA with traditional point‐of‐care testing (POCT) techniques, including enzyme‐linked immunosorbent assay (ELISA) and AuNP‐based colorimetric ICA, in pathogen detection. As shown in Figure 4n, the LOD of commercial ELISA kit for SARS‐CoV‐2 SP is 70 pg mL^−1^, which is 58 times higher than that of our method. Notably, ELISA procedures are complex and time‐consuming (≈2–3 h), while our proposed FeAu@AuIr‐ICA is simple to operate and only takes 20 min. Therefore, our proposed nanozyme‐ICA offers better user‐friendliness and on‐site rapid testing capabilities. Moreover, compared to the current mainstream AuNP‐based ICA, the multi‐tentacle FeAu@AuIr nanozyme‐mediated ICA possesses higher sensitivity, broader detection range, better quantification capability, and more detection channels. The results in Figure 4o show that the visual LOD of AuNP‐ICA for SARS‐CoV‐2 SP, *P. aeruginosa*, and *S. pneumoniae* are 0.5 ng mL^−1^, 5 × 10^3^ cells mL^−1^, and 5 × 10^3^ cells mL^−1^, respectively. Therefore, the sensitivity of our proposed FeAu@AuIr‐ICA in catalytic mode is improved by at least 238 times compared to AuNP‐ICA, with a detection range increased by more than two orders of magnitude. In addition, we also compared the overall performance of the FeAu@AuIr‐based nanozyme‐ICA with the recently reported ICA approaches for detection of respiratory pathogens (Table S1, Supporting information). Evidently, only our method, which is based on the universal recognition capabilities of MPBA and antibody‐modified test strips, can simultaneously detect respiratory bacteria and virus with high sensitivity. Furthermore, FeAu@AuIr‐ICA demonstrates better versatility, higher scalability, and stronger detection throughput compared to other ICA methods, without requiring complex detection instruments. These findings clearly demonstrate the obvious advantages of our multi‐tentacle nanozyme‐ICA in POCT applications, particularly its superior performance compared to ELISA and current ICA methods.

### 2.4 Application of Universal Nanozyme‐ICA for Real Clinical Specimens

**Figure 5:**
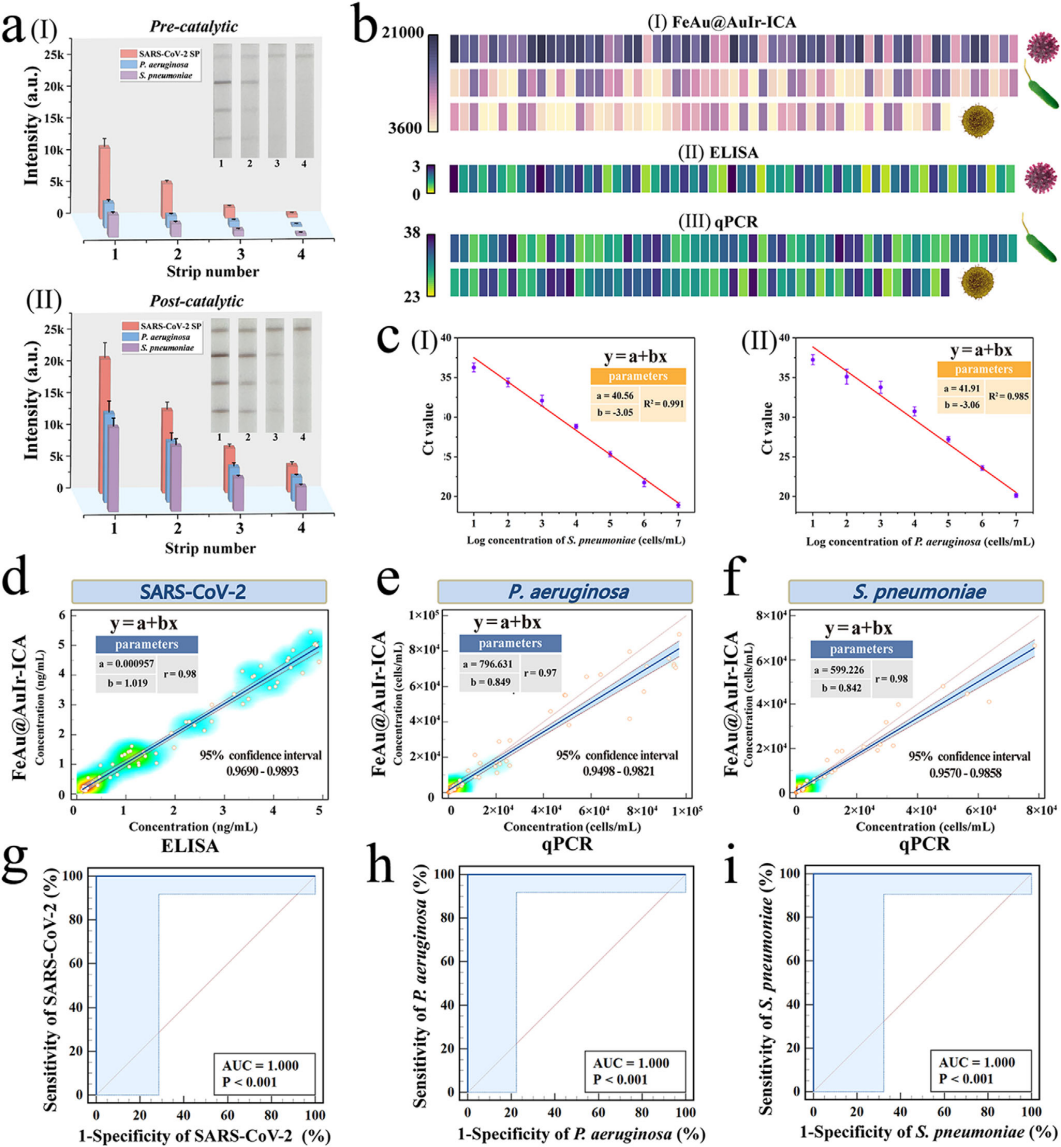
Application of FeAu@AuIr‐based nanozyme‐ICA for real clinical samples detection. a) Results of the FeAu@AuIr‐ICA on swab samples spiked with pathogens before catalysis (I) and after catalysis (II). b) T line colorimetric signal on FeAu@AuIr‐ICA strips (catalytic mode) (I), OD value of ELISA (II), and Ct value of qPCR (III) for the detection of clinical positive specimens. c) Standard curve of qPCR for detection of *S. pneumoniae* (I) and *P. aeruginosa* (II). d) Deming regression analysis results on SARS‐CoV‐2 in positive throat swab specimens detected by FeAu@AuIr‐ICA and ELISA. Deming regression analysis results on *P. aeruginosa* e) and *S. pneumoniae* f) in positive bronchoalveolar lavage fluid samples detected by FeAu@AuIr‐ICA and qPCR. ROC curve of the prediction models for the diagnosis of g) SARS‐CoV‐2, h) *P. aeruginosa*, and i) *S. pneumoniae* infections based on the proposed nanozyme‐ICA.

The three respiratory pathogens (*S. pneumoniae*, *P. aeruginosa*, SARS‐CoV‐2) targeted in this study are important causative agents of current respiratory infections and community acquired pneumonia. Through spiking experiments with throat swab samples, we first verified the capability of our universal nanozyme‐ICA to simultaneously monitor respiratory virus and bacteria in actual clinical applications. **Figure** **5**a(I,II) shows the detection results of the universal nanozyme‐ICA based on FeAu@AuIr‐MPBA probe for target virus/bacteria in throat swab samples high concentration (10 ng mL^−1^; 10^5^ cells mL^−1^), medium concentration (1 ng mL^−1^; 10^4^ cells mL^−1^), medium‐low concentration (0.1 ng mL^−1^; 10^3^ cells mL^−1^), and low concentration (0.01 ng mL^−1^; 10^2^ cells mL^−1^), before and after catalysis, respectively. The colorimetric signals and catalytic enhanced signals on the T‐line of the test strip remained stable in real respiratory samples and were consistent with laboratory system results. By quantifying the colorimetric signals on the T‐line before and after catalysis using ImageJ software and applying them to the pre‐established standard calibration curve (Figure 4c,d), the average recovery rates of FeAu@AuIr‐ICA for target pathogens before catalysis ranged from 86.19% to 115.94%, with relative standard deviations (RSD) ≤ 11.83% (Table S2, Supporting information). Meanwhile, under catalytic mode, the average recovery rate of FeAu@AuIr‐ICA for the target pathogens was between 87.08% and 111.00%, with an RSD ≤ 12.69% (Table S3, Supporting information). It should be noted that before the catalytic reaction, the colorimetric signal of T‐lines produced by low‐concentration pathogens (bacteria<10^2 ^cells mL^−1^; virus<0.01 ng mL^−1^) cannot be accurately quantified using the standard curve. However, after the catalytic reaction, the detection range of the test strip is significantly expanded, enabling accurate detection of pathogens at all concentration levels. The results indicate that the FeAu@AuIr‐MPBA‐based nanozyme‐ICA can stably and accurately quantify pathogens in complex real samples, and the catalytic mode can further enhance the sensitivity and detection range of the proposed assay.

Subsequently, we utilized the established FeAu@AuIr‐ICA for the rapid diagnosis of respiratory bacteria/virus from clinical nasopharyngeal swab specimens and bronchoalveolar lavage fluid of infected patients, to further validate its effectiveness in clinical applications. We collected 59 SARS‐CoV‐2‐positive throat swab samples, 59 *P. aeruginosa*‐positive bronchoalveolar lavage samples, 52 *S. pneumoniae*‐positive bronchoalveolar lavage samples, 15 target pathogen‐negative throat swab samples, and 20 target pathogen‐negative bronchoalveolar lavage samples from the Department of Laboratory Medicine of Guangdong Provincial People's Hospital, under the guidance of the hospital's ethics committee (approval number: KY2024‐678‐02). Before detection, we validated these clinical samples using real‐time quantitative PCR (qPCR) or ELISA (experimental procedures are detailed in Section S1.7, Supporting Information). Then, we plotted the standard curves for the qPCR method based on the relationship between the concentrations of *S. pneumoniae* (I) and *P. aeruginosa* (II) and their Ct values (Figure 5c). Figure 5b‐(I–III) show the detection results (colorimetric signal intensity of T‐line) of positive clinical samples using FeAu@AuIr‐ICA test strips under catalytic enhancement mode, the OD value intensity of SARS‐CoV‐2‐positive throat swab samples detected by ELISA, and the Ct values of *P. aeruginosa* and *S. pneumoniae* positive samples detected by qPCR, respectively. The data from the nanozyme‐ICA strips, ELISA, and qPCR were substituted into the corresponding standard curves (Figures 4c–e,n and 5c) to calculate the pathogen concentrations obtained by different methods. Table S4 (Supporting information) shows the detection results of FeAu@AuIr‐ICA for SARS‐CoV‐2 positive and negative samples. Compared with the results obtained from ELISA kits, the accuracy of our nanozyme‐ICA completely matches that of ELISA, but has a notably higher detection range for high viral concentrations. In addition, the detection results shown in Table S5 (Supporting information) demonstrate that the nanozyme‐ICA based on FeAu@AuIr‐MPBA can accurately diagnose target bacteria (*P. aeruginosa* and *S. pneumoniae*) in bronchoalveolar lavage samples, achieving complete consistency with qPCR results in both positive and negative specimens. The Deming regression lines in Figure 5d–f indicate that the slopes for SARS‐CoV‐2, *P. aeruginosa*, and *S. pneumoniae* are all close to 1 (SARS‐CoV‐2: 1.019, *P. aeruginosa*: 0.849, *S. pneumoniae*: 0.842), with 95% confidence intervals of 0.9690–0.9893, 0.9498–0.9821, and 0.9570–0.9858, respectively. The receiver operating characteristic（ROC）curves in Figure 5g–i further demonstrate that our method has 100% sensitivity and specificity for real clinical specimens. Notably, conventional microbial plate culture for pathogenic microorganisms typically requires 12 to 48 h, and rapid detection methods such as qPCR and ELISA need 2–3 h of testing time. In contrast, our method requires only 20 min (5 min for pathogen capture and enrichment, 13 min for chromatography, and 2 min for the catalytic reaction) to perform universal detection of bacteria and virus. To the best of our knowledge, this work represents the first successful development of a high‐performance ICA platform for the simultaneous detection of bacteria and virus using a universal recognition molecule. The broad‐spectrum and highly sensitive detection capabilities of the MPBA‐modified multi‐tentacle magnetic nanozyme make it a promising candidate for application to other infectious pathogens, including foodborne and intestinal microorganisms as well as emerging pathogens. We believe this technology has the potential to replace traditional double‐antibody sandwich ICA methods and emerge as a new‐generation POCT technique for pathogenic microorganisms.

## 3 Conclusion

In summary, we achieved a breakthrough in rapid on‐site detection of respiratory pathogens through the development of MPBA‐mediated magnetic nanozyme‐ICA method. Through this technology, a universal ICA platform for combined testing of respiratory bacteria and virus was established, successfully achieving rapid, ultrasensitive, and simultaneous screening of *S. pneumoniae*, *P. aeruginosa*, and SARS‐CoV‐2 in clinical samples. Our newly developed biomimetic multi‐tentacle FeAu@AuIr‐MPBA probe consists of a 160 nm Fe_3_O_4_ magnetic core, a layer of large Au_15_ NPs, a layer of smaller AuIr_5_ catalytic particles, and surface‐modified MPBA molecules from inside to outside. It provides both broad‐spectrum capture capability and high magnetic responsiveness for rapid bacterial/viral enrichment, as well as strong colorimetric/catalytic activity for immunochromatographic detection. Experimental results demonstrate that FeAu@AuIr‐MPBA can efficiently capture target pathogens within 5 min (capture efficiency >87.83%), complete chromatographic detection in 13 min, and catalytic enhancement in 2 min, all within a total assay time of 20 min. In direct detection mode, the assay achieved LODs of 993 cells mL^−1^ for *S. pneumoniae*, 1257 cells mL^−1^ for *P. aeruginosa*, and 20.0 pg mL^−1^ for SARS‐CoV‐2. Following catalytic enhancement, the LODs were further reduced to 21 cells mL^−1^ for *S. pneumoniae*, 17 cells mL^−1^ for *P. aeruginosa*, and 1.2 pg mL^−1^ for SARS‐CoV‐2. Moreover, the catalytic mode of the FeAu@AuIr‐ICA exhibits ≈58 times higher sensitivity than traditional ELISA and 238–416 times higher sensitivity than AuNP‐based colorimetric ICA. The accuracy and reliability of the FeAu@AuIr‐ICA were further validated in clinical applications by testing 59 SARS‐CoV‐2–positive samples, 59 *P. aeruginosa*–positive samples, and 52 *S. pneumoniae*–positive samples, confirming that the assay results are consistent with those obtained using ELISA and qPCR. To our knowledge, this work is the first to utilize universal recognition molecule for the simultaneous detection of bacteria and virus in ICA method. The FeAu@AuIr‐ICA supports multi‐channel detection, enabling combined testing of multiple respiratory pathogens on a single test strip, meeting the high clinical demand for POCT technology. More importantly, this technology can be further upgraded into a universal detection tool for other infectious disease pathogens by replacing antibodies on the test strips, thus enabling broader applications in public health surveillance and clinical diagnosis.

## 4 Experimental Section

### Reagents and Instruments

Ferric chloride (FeCl_3_·6H_2_O), PEI (MW 25 kDa), chloroauric acid tetrahydrate (HAuCl_4_·4H_2_O), fetal bovine serum (FBS), Bovine serum albumin (BSA), 5,5′ ‐dithiobis (2‐nitrobenzoic acid) (DTNB), 4‐MPBA, H_2_O_2_, TMB, AEC, PBS, and Tween‐20 were supplied by Sigma‐Aldrich (USA). anti‐SARS‐CoV‐2 S1 protein antibody (Catalog# 40591‐MM43) was purchased from Sino Biological Inc. (Beijing, China). Anti‐*P. aeruginosa* antibody (Catalog# MA1‐83430), anti‐*S. pneumoniae* antibody (Catalog# rmAb‐Strpcpnu‐001) were purchased from Jiangnan University. (Jiangsu, China). ICA components, including NC membrane (CN95), sample pad, absorbent pad, and plastic plate were obtained from Jieyi Biotechnology Co., Ltd. (Shanghai, China).

The morphology characterization of the synthesized nanomaterials was performed using an FEI Tecnai G2 F20 TEM with an acceleration voltage of 200 kV and a JEOL JSM‐7001F SEM with an acceleration voltage of 10 kV. The zeta potential of the products at each stage was analyzed using a ZS90 zeta analyzer (Malvern, UK). The magnetic properties of synthetic magnetic nanozymes were studied using a superconducting quantum interference device magnetometer (MPMSXL‐7, USA) at 300 K. NTA was performed using a Zetaview‐PMX120‐Z (Particle Metrix, Meerbusch, Germany) to determine the concentration of spherical nanozyme particles.

### Clinical Sample Collection

All human respiratory specimens used in this study, including throat swab samples (59 SARS‐CoV‐2 positive throat swabs and 15 target pathogen‐negative throat swabs) and bronchoalveolar lavage fluid samples (59 *P. aeruginosa‐*positive bronchoalveolar lavage fluid samples, 52 *S. pneumoniae*‐positive bronchoalveolar lavage fluid samples, and 20 target pathogen‐negative bronchoalveolar lavage fluid samples), were obtained from the Department of Laboratory Medicine of Guangdong Provincial People's Hospital with approval from the Hospital (Approval number: KY2024‐678‐02). All patients and healthy volunteers have signed informed consent forms agreeing to participate in sample collection.

### Preparation of Multi‐Tentacle FeAu@AuIr Nanozyme

First, 5 mg of Fe_3_O_4_ powder was dissolved in 40 mL of deionized water and ultrasonicated for 5 min to ensure complete dispersion. Subsequently, 5 mL of PEI (0.5 mg mL^−1^) aqueous solution was added, and ultrasonication was continued for 30 minutes to uniformly coat the Fe_3_O_4_ surface with a positively charged PEI shell, converting the Fe_3_O_4_ solution from negatively charged to positively charged. After ultrasonication, magnetic separation was used to remove the supernatant, and the precipitate was washed three times with deionized water to remove excess PEI. The resulting precipitate was dispersed in 5 mL of deionized water to form a Fe_3_O_4_‐PEI aqueous solution. Next, 40 mL of 15 nm AuNPs was added to the Fe₃O₄‐PEI solution, followed by vigorous ultrasonication for 30 minutes. This process enabled the negatively charged AuNPs to self‐assemble onto the positively charged Fe₃O₄‐PEI surface, forming the FeAu nanocomposite. The FeAu solution was then diluted to 40 mL, and 2 mL of PEI aqueous solution (0.5 mg mL^−1^) was added. The mixture was ultrasonicated vigorously for another 30 min to form FeAu‐PEI. Following this, 30 mL of 5 nm AuIr NPs was added to the FeAu‐PEI solution, and ultrasonication was continued for 20 min to uniformly adsorb AuIr_5_ onto the FeAu surface. The mixture was washed once with deionized water to remove any nonmagnetic NPs. Finally, the resulting FeAu@AuIr MNPs were dispersed in 20 mL of ethanol and stored at 4 °C in the dark for further use.

MPBA modification and carboxylation of the FeAu@AuIr surface can be easily achieved by mixing FeAu@AuIr ethanol solution (10 mL) with freshly prepared MPBA (100 µL, 10 mm) and DTNB (100 µL, 10 mm) respectively, followed by reaction under intense sonication for 1 h. After washing with ethanol and storing in PBS buffer, FeAu@AuIr‐MPBA and FeAu@AuIr‐DTNB can be used directly.

### Preparation of Multi‐Channel ICA Strip

Using a spray dispenser (Biodot XYZ5050), streptavidin (1.0 mg mL^−1^), anti‐SARS‐CoV‐2 S1 protein antibodies (1.0 mg mL^−1^), anti‐P*. aeruginosa* antibody (1.2 mg mL^−1^), and anti‐*S. pneumoniae* antibody (1.2 mg mL^−1^) were sequentially sprayed onto the CN95 NC membrane at a rate of 0.8 µL cm⁻¹. The NC membrane was then placed in a forced‐air oven at 35 °C and dried for 4 h. The dried NC membrane was assembled with a PVC backing plate, sample pad, and absorbent pad to form the ICA test strip. Finally, the assembled strips were cut into 3 mm widths and stored in a drying chamber for subsequent use.

### Procedure for Simultaneously Detecting Three Pathogens

First, a series of pathogen mixture solutions were prepared containing different concentrations of *S. pneumoniae* (10⁵–10 cells mL^−1^), *P. aeruginosa* (10⁵‐10 cells mL^−1^), and SARS‐CoV‐2 SP (10‐0.001 ng mL^−1^). Subsequently, 4 µL of FeAu@AuIr‐MPBA and 1 µL of C‐line supplement nanolabels (FeAu@AuIr‐biotin) were added to each test sample. The mixture was vortexed and incubated for 5 min, followed by magnetic enrichment of the complexes using an external magnetic field. The supernatant was discarded, and the complexes were resuspended in 90 µL of running buffer (1% PBST + 5% milk). The prepared solution was added to ICA strips, and chromatographic reaction was carried out for 13 min. Afterward, the test strips were placed on a whiteboard for visual observation and photographed using a mobile phone. Subsequently, 6 µL of catalytic solution (H₂O₂:AEC = 4:6) was evenly applied to the NC membrane. After 2 min, a second photograph was taken for further analysis. ImageJ software was used to quantify the signal intensity before and after catalysis. The LODs for *S. pneumoniae*, *P. aeruginosa*, and SARS‐CoV‐2 SP were calculated based on the grayscale value of the negative control plus three standard deviations.

In this study, ImageJ software was used to analyze the grayscale values of ICA test strips. First, the color images of the test strips were converted to an 8‐bit format. Then, a rectangular tool was used to draw a selection area on the T‐line of the test strip, ensuring that the selection area completely covered the T‐line while avoiding background interference. Next, the measurement function was used to obtain grayscale value data for each selected area, and the results were recorded for further analysis. To eliminate background effects, the grayscale values of the background area were also measured and corrected.

### Statistical Analysis

Origin 2021 software (OriginLab Corporation, USA) was used for graphical representation and statistical analysis. Data shown in figures were expressed as mean ± standard deviation (SD) from three independent replicate experiments (n = 3). MedCalc software (MedCalc Software Ltd., Belgium) was used for Deming regression analysis and ROC curve analysis to evaluate the consistency between FeAu@AuIr‐ICA and ELISA/qPCR, as well as the specificity of the test strips, with sample sizes indicated in the Figure legends. Advantage software (Thermo Fisher Scientific, USA) was used for graphical representation and statistical analysis. XPS data was processed using Advantage software (Thermo Fisher Scientific, USA), utilizing the software's built‐in peak fitting functions for peak identification and deconvolution.

## Conflict of Interest

The authors declare no conflict of interest.

## Author Contributions

Q.Y. and J.X.L. contributed equally to this work. C.W.W., S.Z., and B.G. designed and managed the study. Q.Y., J.X.L., S.Z., Y.J.H., B.S.T. and M.R.L. performed the experiments. Q.Y. and J.X.L. analyzed the experimental data. C.W.W., Q.Y., and J.X.L. wrote the manuscript. All authors reviewed the manuscript.

## Supporting information



Supporting Information

## Data Availability

The data that support the findings of this study are available from the corresponding author upon reasonable request.

## References

[1] N. H. L. Leung , Nat. Rev. Microbiol. 2021, 19, 528. 33753932 10.1038/s41579-021-00535-6 PMC7982882

[2] W. H. Man , M. A. van Houten , M. E. Mérelle , A. M. Vlieger , M. L. J. N. Chu , N. J. G. Jansen , E. A. M. Sanders , D. Bogaert , Lancet. Respir. Med. 2019, 7, 417. 30885620 10.1016/S2213-2600(18)30449-1 PMC7172745

[3] S. Belman , N. Lefrancq , S. Nzenze , S. Downs , M. du Plessis , S. W. Lo , L. McGee , S. A. Madhi , A. von Gottberg , S. D. Bentley , H. Salje , Nature 2024, 631, 386. 38961295 10.1038/s41586-024-07626-3 PMC11236706

[4] M. H. J. Rhodin , A. C. Reyes , A. Balakrishnan , N. Bisht , N. M. Kelly , J. S. Gibbons , J. Lloyd , M. Vaine , T. Cressey , M. Crepeau , R. Shen , N. Manalo , J. Castillo , R. E. Levene , D. Leonard , T. Zang , L. Jiang , K. Daniels , R. M. Cox , C. M. Lieber , J. D. Wolf , R. K. Plemper , S. R. Leist , T. Scobey , R. S. Baric , G. Wang , B. Goodwin , Y. S. Or , Nat Commun. 2024, 15, 6503. 39090095 10.1038/s41467-024-50931-8 PMC11294338

[5] a) G. B. D. Diseases , C. Injuries , Lancet 2020, 396, 1204; 33069326 10.1016/S0140-6736(20)30925-9 PMC7567026

[6] Antimicrobial Resistance Collaborators , Lancet Glob Health. 2024, 12, e201.38134946

[7] a) M. Xiao , F. Tian , X. Liu , Q. Zhou , J. Pan , Z. Luo , M. Yang , C. Yi , Adv. Sci. 2022, 9, 2105904; 10.1002/advs.202105904 PMC9110880 35393791

[8] a) Z. Zhang , P. Ma , R. Ahmed , J. Wang , D. Akin , F. Soto , B. F. Liu , P. Li , U. Demirci , Adv. Mater. 2022, 34, 2103646; 10.1002/adma.202103646 34623709

[9] J. Shen , X. Zhou , Y. Shan , H. Yue , R. Huang , J. Hu , D. Xing , Nat. Commun. 2020, 11, 267. 31937772 10.1038/s41467-019-14135-9 PMC6959245

[10] a) G. Zhang , T. Liu , H. Cai , Y. Hu , Z. Zhang , M. Huang , J. Peng , W. Lai , ACS Nano 2024, 18, 2346; 38181225 10.1021/acsnano.3c10427

[11] C. Parolo , A. Sena‐Torralba , J. F. Bergua , E. Calucho , C. Fuentes‐Chust , L. Hu , L. Rivas , R. Álvarez‐Diduk , E. P. Nguyen , S. Cinti , D. Quesada‐González , A. Merkoçi , Nat. Protoc. 2020, 15, 3788. 33097926 10.1038/s41596-020-0357-x

[12] a) X. Huang , L. Chen , W. Zhi , R. Zeng , G. Ji , H. Cai , J. Xu , J. Wang , S. Chen , Y. Tang , J. Zhang , H. Zhou , P. Sun , Anal. Chem. 2023, 95, 13101; 37526338 10.1021/acs.analchem.3c01631

[13] a) X. Liu , Y. Chen , T. Bu , Z. Deng , L. Zhao , Y. Tian , C. Jia , Y. Li , R. Wang , J. Wang , D. Zhang , Biosens. Bioelectron. 2023, 229, 115239; 36965382 10.1016/j.bios.2023.115239

[14] a) S. Liu , R. Shu , J. Ma , L. Dou , W. Zhang , S. Wang , Y. Ji , Y. Li , J. Xu , D. Zhang , M. Zhu , Y. Song , J. Wang , Chem Eng J. 2022, 446, 137382;

[15] a) R. Chen , X. Chen , Y. Zhou , T. Lin , Y. Leng , X. Huang , Y. Xiong , ACS Nano 2022, 16, 3351; 35137583 10.1021/acsnano.2c00008

[16] a) P. Wu , W. Zuo , Y. Wang , Q. Yuan , J. Yang , X. Liu , H. Jiang , J. Dai , F. Xue , Y. Ju , Chem. Eng. J. 2023, 451, 139021;

[17] C. Wang , Q. Yu , S. Zheng , W. Shen , J. Li , C. Xu , B. Gu , ACS Nano 2024, 18, 16752. 38901038 10.1021/acsnano.4c01824

[18] a) C. Wang , X. Yang , B. Gu , H. Liu , Z. Zhou , L. Shi , X. Cheng , S. Wang , Anal. Chem. 2020, 92, 15542; 33207872 10.1021/acs.analchem.0c03484

[19] Q. Xue , H.‐Y. Sun , Y.‐N. Li , M.‐J. Zhong , F.‐M. Li , X. Tian , P. Chen , S.‐B. Yin , Y. Chen , Chem Eng J. 2021, 421, 129760.

[20] M. Zhao , X. Yao , J. Li , H. Hu , J. Ren , J. Xu , J. Wang , D. Zhang , Biosens. Bioelectron. 2023, 230, 115264. 37004282 10.1016/j.bios.2023.115264

[21] a) T. Bai , L. Wang , M. Wang , Y. Zhu , W. Li , Z. Guo , Y. Zhang , Biosens. Bioelectron. 2022, 208, 114218; 35358773 10.1016/j.bios.2022.114218

[22] a) J. Feng , X. Yang , T. Du , L. Zhang , P. Zhang , J. Zhuo , L. Luo , H. Sun , Y. Han , L. Liu , Y. Shen , J. Wang , W. Zhang , Adv. Sci. 2023, 10, 2303078; 10.1002/advs.202303078 PMC10667809 37870181

[23] X. Chen , X. Wang , Y. Fang , L. Zhang , M. Zhao , Y. Liu , Anal. Chem. 2022, 94, 8382. 35647701 10.1021/acs.analchem.2c00877

[24] G. Huang , H. Zhao , P. Li , J. Liu , S. Chen , M. Ge , M. Qin , G. Zhou , Y. Wang , S. Li , Y. Cheng , Q. Huang , J. Wang , H. Wang , L. Yang , Anal. Chem. 2021, 93, 16086. 34730332 10.1021/acs.analchem.1c03807

[25] X. Su , X. Liu , Y. Xie , M. Chen , C. Zheng , H. Zhong , M. Li , ACS Nano 2023, 17, 4077. 36758150 10.1021/acsnano.3c00449

