# Supplementary material for: Mercaptophenylboronic Acid‐Mediated Nanozyme Immunochromatographic Assay for Simultaneous Detection of Respiratory Bacteria and Virus
Source: Adv Sci (Weinh). 2025 May 8;12(31):2502574. doi: 10.1002/advs.202502574 (PMC12376685; doi:10.1002/advs.202502574)
Supplement: Supplementary file 1 — Supporting Information [file ADVS-12-2502574-s001.docx]

Supporting Information

**Mercaptophenylboronic acid-Mediated Nanozyme Immunochromatographic Assay for Simultaneous Detection of Respiratory Bacteria and Virus**

Qing Yu ^a,b^, Jiaxuan Li ^a,b^, Shuai Zheng ^c,^*, Yajin Hu ^b^, Benshun Tian ^b^, Meirou Lu ^a,b^, Bing Gu ^a,b,^*, Chongwen Wang ^a,b,^*

^a^ Department of Clinical Laboratory Medicine, Guangdong Provincial People’s Hospital (Guangdong Academy of Medical Sciences), Southern Medical University, Guangzhou, Guangdong 510000, China

^b^ School of Medicine, South China University of Technology, Guangzhou, 510006, China

*^*^*Corresponding author

^c^ Hefei Institute of Physical Science, Chinese Academy of Sciences, Hefei 230036, China.

**Supporting Material Contents.**

S1. Supplementary experimental section (S.1.1-S.1.11)

S2 Calculation of AuIr loading on FeAu@AuIr and Fe@AuIr MNPs

S3 Optimization of the FeAu@AuIr nanozyme size

Supplement Figures S1-S17

Tables S1-S5

**S1 Experimental section**

**S1.1 Preparation of 160 nm Fe_3_O_4_**

Magnetic Fe_3_O_4_ MNPs with a diameter of 160 nm were synthesized via a modified solvothermal method using a dual-solvent system. Specifically, 60 mg of FeCl₃·6H₂O was dissolved in a 20 mL mixed solvent of diethylene glycol (DEG) and ethylene glycol (EG) (V_EG_:V_DEG_ = 1:4). Subsequently, 2.5 g of polyvinylpyrrolidone (PVP) was added, and the mixture was heated and stirred in an oil bath at 120°C until the PVP was completely dissolved. Next, 2 g of anhydrous sodium acetate (NaOAc) powder was introduced into the solution, followed by stirring for 30 min to ensure the solution is completely transparent. The resulting solution was then transferred into a Teflon-lined autoclave and subjected to a solvothermal reaction at 210°C for 12 h in a forced-air oven. The obtained black precipitate was washed twice with deionized water and ethanol, respectively, and dried at 60°C in a vacuum oven. The final product was 160 nm Fe_3_O_4_ MNPs.

**S1.2 Preparation of 3 nm Au NPs**

First, 100 mL of deionized water, 1 mL of 1% (w/v) chloroauric acid (HAuCl₄), and 1 mL of 1% (w/v) trisodium citrate (NaCit) were added to a clean Erlenmeyer flask. After thorough mixing, 3 mL of sodium borohydride (NaBH₄, 100 mM) was introduced into the solution. The mixture was stirred continuously for 4 h. The resulting product was stored in the dark for subsequent use.

**S1.3 Preparation of 15 nm Au NPs (Au_15_)**

Au_15_ NPs with a diameter of 15 nm were synthesized using the citrate reduction method. Briefly, 100 mL of deionized water and 1 mL of 1% (w/v) HAuCl₄ were added to a clean Erlenmeyer flask, and the mixture was stirred thoroughly before being heated to boiling. Subsequently, 2.5 mL of 1% (w/v) trisodium citrate (NaCit) was added in a single step. The solution was vigorously stirred for 15 min, after which the heating was turned off. Stirring was continued until the mixture reached room temperature. The final product was stored in the dark for subsequent use.

**S1.4 Preparation of AuIr_5_ NPs**

5 nm AuIr NPs were synthesized via the seed-mediated growth method. Specifically, 200 mL of pre-prepared 3 nm Au NPs, 12 mL of 1% NaCit (w/v), and 4 mL of Na₂IrCl₆·6H₂O (2 M) were added to a clean Erlenmeyer flask. The mixture was preheated to 80°C using an oil bath, followed by the addition of 2 mL NaBH₄ (100 mM). After stirring for 1 h, the heating was turned off, and the reaction was allowed to proceed with stirring for an additional 12 h. The resulting product was stored in the dark for subsequent use.

**S1.5 Preparation of C line tag and immuno-FeAu@AuIr tags**

A 1 mL aliquot of FeAu@AuIr-DTNB was washed once with ethanol and PBST to remove unbound DTNB molecules and then redispersed in 500 μL of MES buffer (100 mM, pH 5.5). Subsequently, freshly prepared EDC (5 μL, 0.1 M) and sulfo-NHS (10 μL, 0.1 M) were added, and the mixture was ultrasonicated for 10 min to activate the carboxyl groups on the DTNB-modified FeAu@AuIr.

After washing once with PBST, the magnetic particles were redispersed in 200 μL of PBST and incubated with 6 μg of biotinylated BSA (BSA-biotin), 6 μg of anti- SARS-CoV-2 SP antibody or 6 μg of anti- *P. aeruginosa* antibody, respectively, for 3 h. To block unreacted carboxyl groups, 80 μL of 10% BSA (w/v) solution was added, followed by an additional 1 h incubation. The product was magnetically separated, washed twice with PBST, and finally redispersed in 300 μL of PBST for subsequent use.

**S1.6 Preparation of AuNP-based colorimetric ICA**

First, AuNPs (20 nm) were fabricated through the citrate reduction method. Briefly, 200 mL of HAuCl_4_ solution (0.01%, w/v) was heated to the boiling point with stirring. Then, 2.2 mL of trisodium citrate (1%, w/v) was added rapidly to the boiling solution. The suspension was boiled for 15 min and then allowed to reach thermal equilibrium at room temperature, which yielded the Au NPs with a diameter of ~20 nm. Afterwards, the pH of 8 μg anti-bacteria antibody or anti-virus antibody was adjusted to 9 with 0.2 M K_2_CO_3_ and incubated with 1 mL 30 nm AuNP (pH 8–9) for 15 min. Then, 50 μL of 10% BSA was added to block the unreacted sites of AuNPs. The as–prepared immuno–AuNPs were collected by centrifugation (8000 rpm, 6 min), and resuspended with 200µL of storage solution (10 mM PB solution containing 1% BSA (w/v), 0.1% PVP (w/v), 10% sucrose (w/v), and 0.05% Tween–20 (v/v)). Finally, it was dispensed onto the glass fiber paper and dried to prepare a conjugate pad. Then, the conjugate pad was assembled on the ICA strip and cut it into a 3.0 mm strip for subsequent use.

**S1.7 Detection of clinical samples**

S.1.7.1 Establishment of standard curve for detecting *P. aeruginosa* and *S. pneumoniae* by qPCR

Firstly, a multiple fluorescence PCR lower respiratory tract bacterial nucleic acid detection kit (Sansure Biotech Inc.) was used to detect standard samples of *P. aeruginosa* and *S. pneumoniae* at different concentrations (10^6^-10^7^ cells/mL). A standard curve for the qPCR method was constructed based on the relationship between the concentration and the Ct value. Secondly, 200 µL of clinical specimens were suspended in 50 μL of nucleic acid extraction solution, transferred to nucleic acid extraction tubes, and then heated at 95°C for 5 min. At this point, nucleic acids from the bacteria were released into the nucleic acid extraction solution. Next, 5 µL of the nucleic acid sample was added to 45 µL of PCR reaction mix and placed into the nucleic acid amplification instrument. The HEX channel was used to detect *S. pneumoniae*, while the CY5 channel was used to detect *P. aeruginosa*. The steps for nucleic acid amplification are as follows:

List 1. qPCR amplification procedure

| **Procedure** | | **Temperature** | **Time** | **Cycle number** |
| --- | --- | --- | --- | --- |
| 1 | UDG enzyme reaction | 50℃ | 2 min | 1 |
| 2 | Predegeneration | 94℃ | 3 min | 1 |
| 3 | Denaturation | 94℃ | 10 s | 45 |
| 4 | Anneal | 60℃ | 20 s |  |
| 5 | Extension and fluorescence detection | 75 ℃ | 20 s |  |
| 6 | Melting curve | 62–75 ℃ | Whole-process acquisition fluorescence | 1 |

Detection result determination:

If the HEX channel detects a typical S-shaped amplification curve with a Ct value <39, the Streptococcus pneumoniae test result is considered positive; if the Ct value >39 or no Ct value is detected, the result is negative.

If the CY5 channel detects a typical S-shaped amplification curve with a Ct value <39, the Pseudomonas aeruginosa test result is considered positive; if the Ct value >39 or no Ct value is detected, the result is negative.

S.1.7.2 Detection of SARS-CoV-2 samples by ELISA

The ELISA kits for the SARS-CoV-2 SP (Catalog #KIT40591) were purchased from Sino Biological, Inc. (Beijing, China). The collected throat swab samples were directly immersed in 1 mL of PBS after collection and tested directly according to the kit instructions.

**S1.8 Calculation of FeAu@AuIr concentration**

The concentration of FeAu@Aulr nanocomposites was determined using Nanoparticle Tracking Analysis (NTA) on the ZetaView-PMX120-Z system (Particle Metrix, Germany), and analyzed using ZetaView software (version 8.05.14SP7). This system measures particle size distribution based on Brownian motion, evaluates particle concentration via video, and enables detection under a fluorescence mode with an excitation wavelength of 520 nm. The sample pool was cleaned with deionized water, calibrated using 100 nm polystyrene microspheres (catalog number: 3100A, Ther-moFisher, USA), and rinsed with 1 × PBS buffer (Biological Industries, Israel). The separated FeAu@Aulr samples were-diluted with 1×PBS buffer according to the experimental requirements (dilution factor specified in the final report) and measured at 11 positions in the sample pool to ensure consistency of results. The experiment was conducted at a controlled temperature of 23-30°C, with continuous monitoring of pH and conductivity. The sample volume prepared was at least 5 mL, and the concentration was optimized according to the experimental requirements to ensure a reliable detection range below 1 μm. Data was recorded and analyzed to determine the particle size distribution and concentration. All experiments were performed according to the manufacturer's operating guidelines, with sample integrity maintained through storage at 4°C and protection from light. The nanoparticle concentration (C) was calculated using the following formula:

$$C=\frac{N}{N_{A}\times V}$$

Where 𝑁 is the number of molecules, 𝑁𝐴 is Avogadro's constant, taken as 6.022×10^23^, and 𝑉 is the volume of the solution (in liters).

**S1.9 DFT calculation**

Density Functional Theory (DFT) calculations were performed using the Vienna Ab Initio Simulation Package (VASP). The Perdew−Burke−Ernzerhof (PBE) generalized gradient approximation (GGA) was used to describe the exchange-correlation potential, the DFT-D3 method was employed to account for van der Waals (vdW) interactions, and the Projector Augmented-Wave (PAW) method was used to calculate the electron-ion interactions. DFT calculations were performed with a cutoff energy of 400 eV, and a 3 × 3 × 1 k-point mesh was used to sample the Brillouin zone. The energy convergence criterion for self-consistent iterations was set to 10^−4^ eV, and the force convergence threshold was set to 0.02 eV/Å. The Gibbs free energy change (ΔG) during the reaction process was calculated using the following formula:

*∆G* =*∆E* +∆ZPE - T*∆S*

Where ΔE is the electron energy difference calculated from DFT, ΔZPE is the zero-point energy difference, T is the room temperature (298.15 K), and ΔS is the entropy change.

**S1.10 FDTD calculation**

The simulations were performed using the fully matched layer (PML) boundary conditions and the ANSYS Lumerical FDTD Solutions for Time-Domain Finite-Difference (FDTD) simulations. The simulation wavelength was set to 500 nm, with the environment having a refractive index of 1 (air). The dielectric constants of Au and Ir were selected from the material database [1] (Front Matter. In Handbook of Optical Constants of Solids, Palik, E. D., Ed. Academic Press: Boston, 1998). The size of the nanoparticles was determined based on experimental TEM measurements. For measurement purposes, the electric field cross-section was monitored. The minimum unit grid size was set to 0.25 nm. The target material structure consisted of a 160 nm Fe_3_O_4_ core with a 15 nm Au nanoparticle layer adsorbed on its surface, with a gap of 15 nm. The outermost layer consisted of a 5 nm AuIr. The electric field cross-section was monitored using a detector, with the minimum unit grid set to 0.25 nm.

**S1.11 Measurement of bacterial cell and viral protein capture efficiency**

The capture efficiency of bacterial cell and viral proteins is evaluated by comparing the changes in bacterial count or protein concentration before and after capture.

(1) Bacterial cell capture efficiency:

A bacterial suspension of known concentration is divided into two groups. One group is directly plated onto agar to determine the initial colony-forming units (CFU) as a control. The other group undergoes treatment with a specific Tag-based capture system, and an equal volume of the supernatant is plated. The bacterial capture efficiency is calculated as follows:

Bacterial cell capture efficiency (%) = (Initial cells−Post-capture cells)/Initial cells × 100%

(2) Viral protein capture efficiency

A viral protein sample of known concentration is divided into two groups. One group is directly analyzed using a BCA protein assay to determine the initial protein concentration (control). The other group is processed with the capture system, and an equal volume of the supernatant is analyzed using the BCA assay to measure residual protein. The viral protein capture efficiency is calculated as follows:

Viral protein capture efficiency (%) = (Initial absorbance - post-capture absorbance) /Initial absorbance × 100%


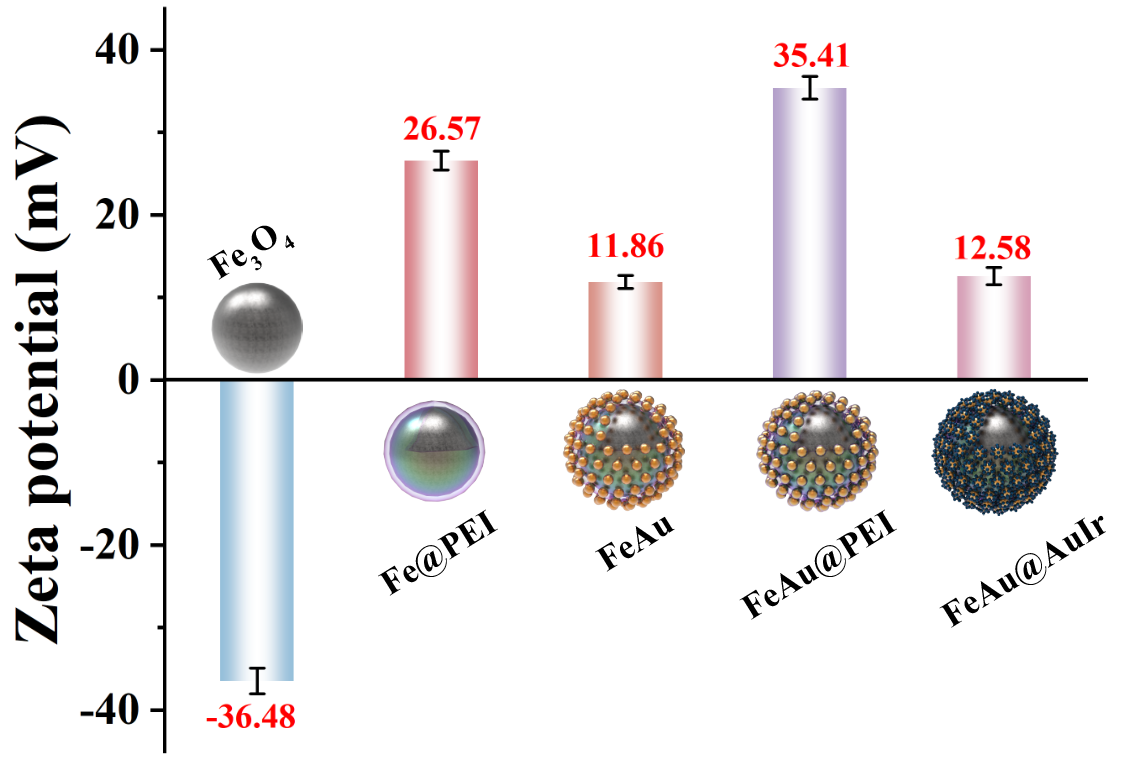


**Figure S1.** Zeta potentials of the prepared FeAu@AuIr at each step.


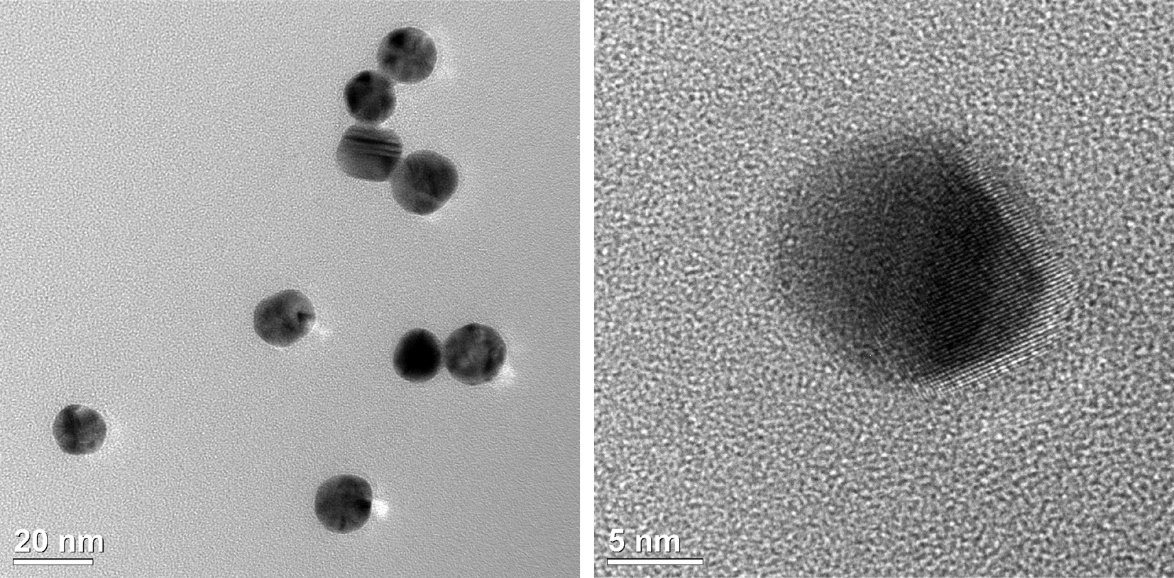


**Figure S2.** TEM images of Au_15_ NPs.


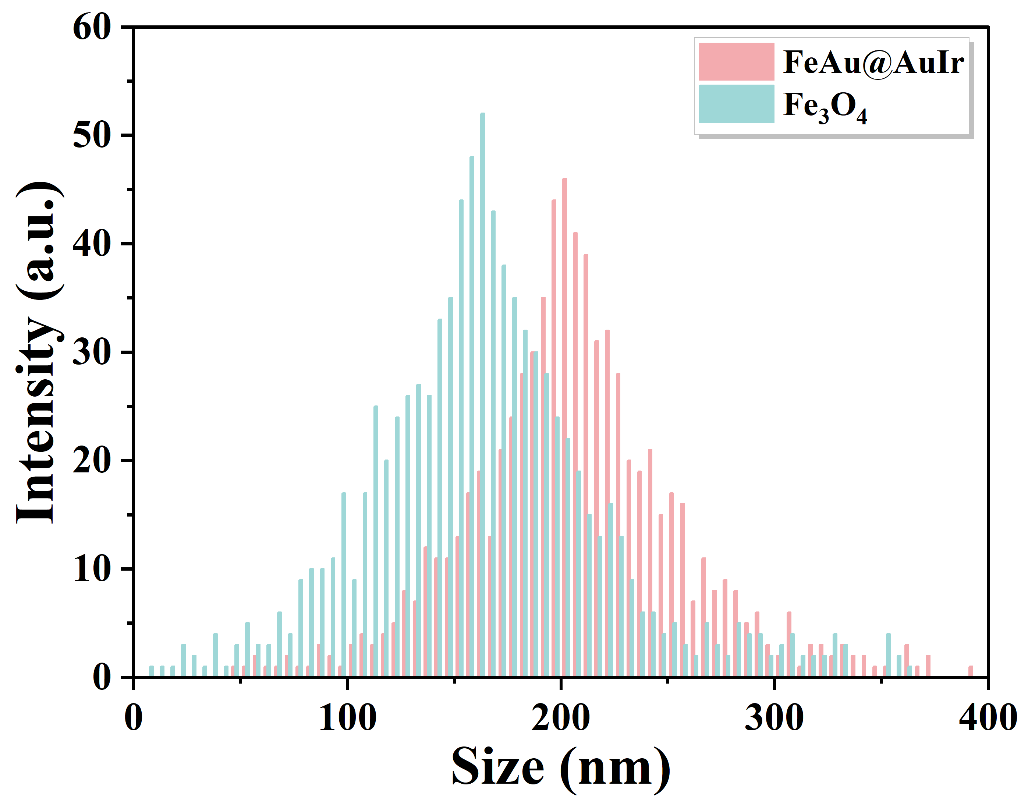


**Figure S3.** Size distribution result of Fe_3_O_4_ and FeAu@AuIr nanozymes from NTA method.

The NTA measurements were conducted using a Zetaview-PMX120-Z device (Particle Metrix, Meerbusch, Germany) and ZetaView software (version 8.05.14 SP7).


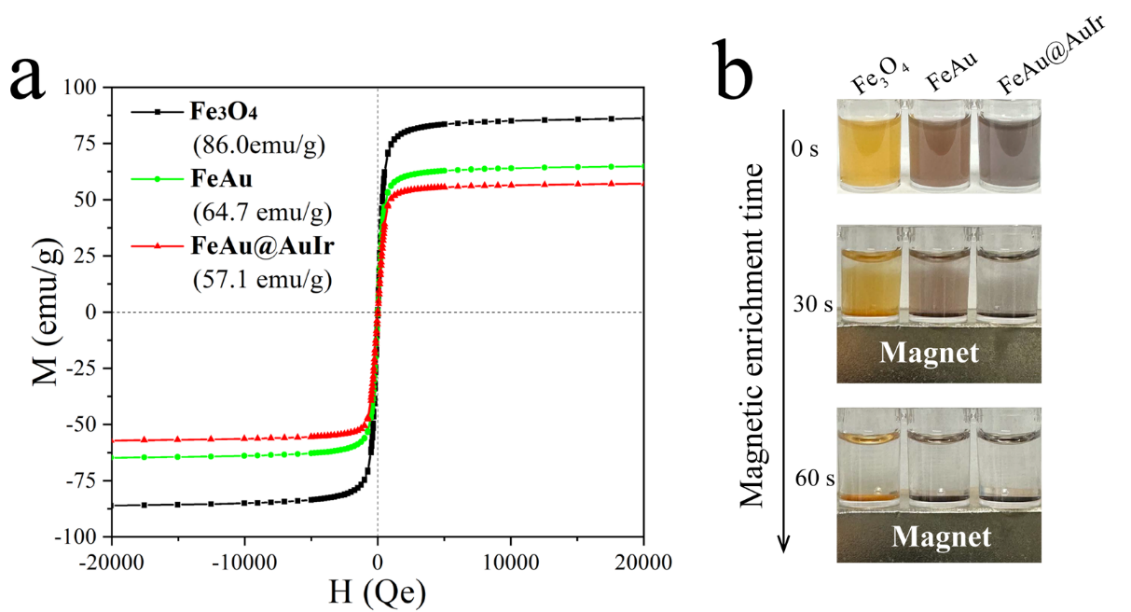


**Figure S4.** (a) Magnetic hysteresis curves and (b) magnetic separation ability of Fe_3_O_4_, FeAu and FeAu@AuIr nanozymes.


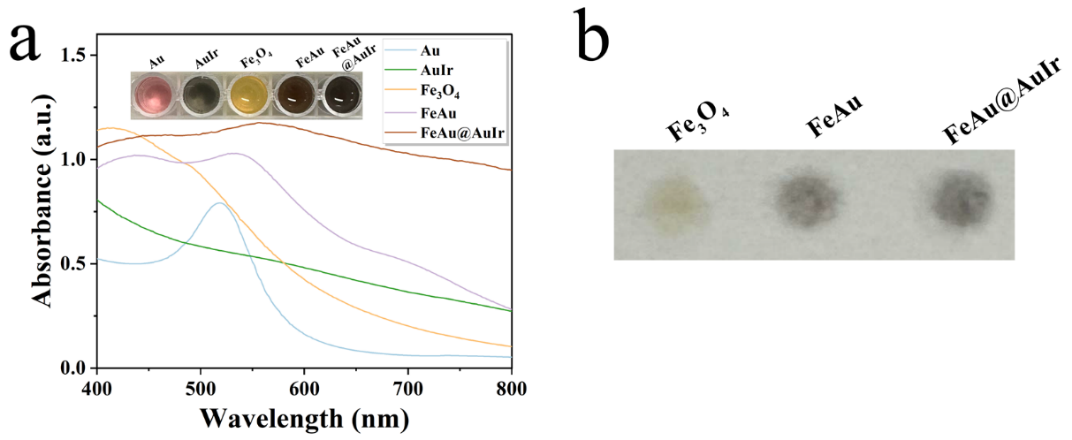


**Figure S5.** (a) UV-vis spectra of Au_15_, AuIr_5_, Fe_3_O_4_, FeAu and FeAu@AuIr. (b) Colorimetric signal of Fe_3_O_4_, FeAu and FeAu@AuIr on NC membrane.


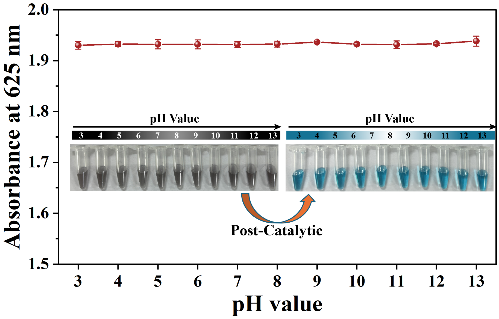


**Figure S6.** Photos and catalytic activities of the FeAu@AuIr nanozyme at different pH values.


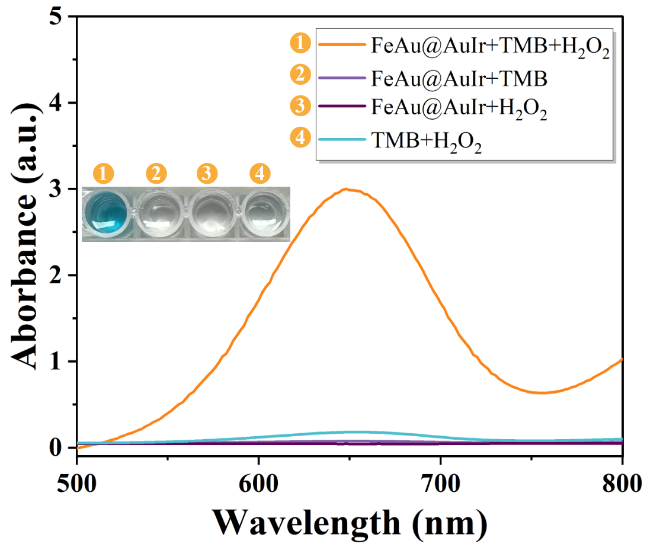


**Figure S7**. Evaluation of the catalytic specificity of the FeAu@AuIr nanozyme toward substrates.


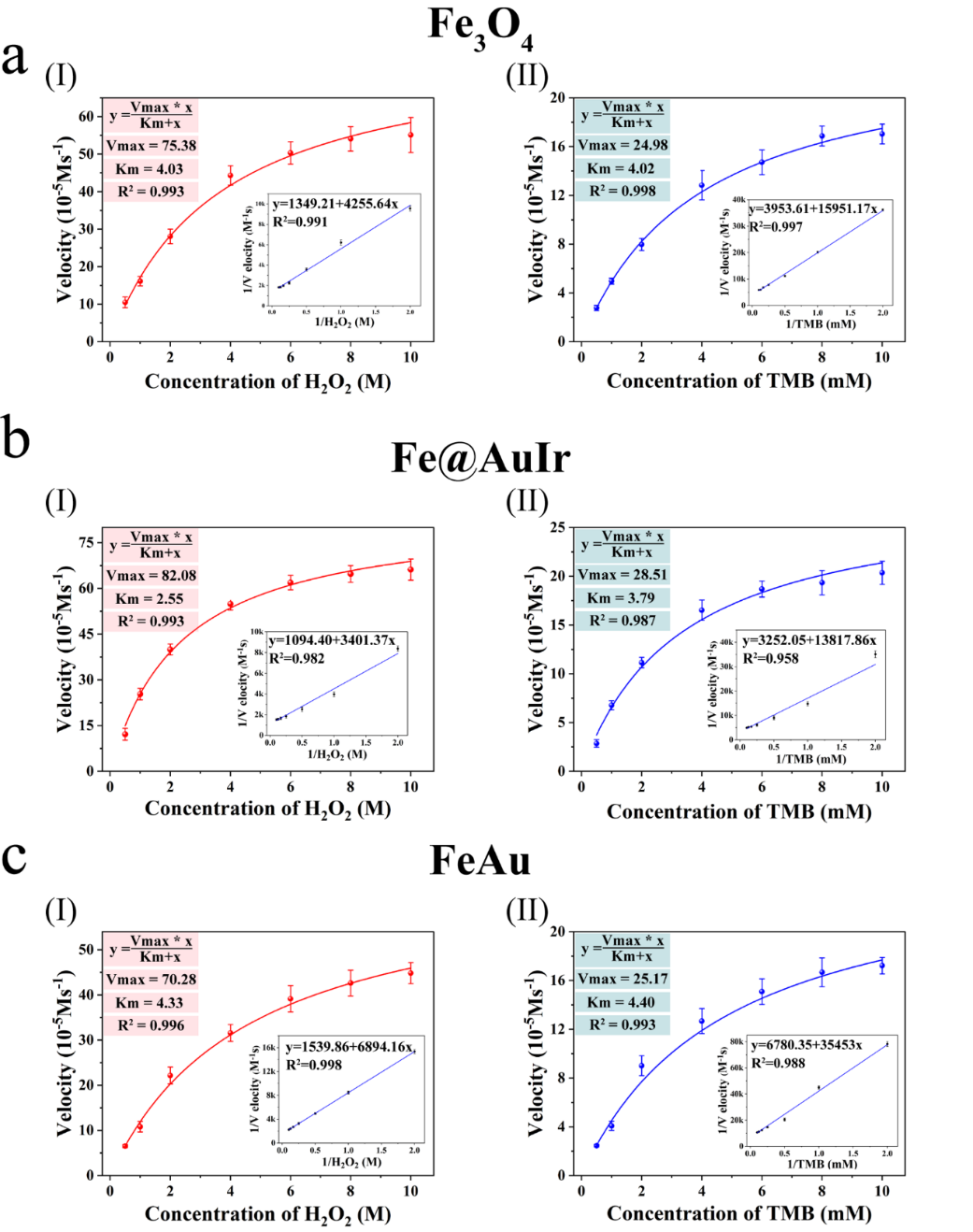


**Figure S8.** Steady-state kinetic analysis of the (a) Fe_3_O_4_, (b) Fe@AuIr, (c) FeAu, (I): plot of reaction velocity versus H_2_O_2_ concentration when TMB concentration is fixed, and (II): plot of reaction velocity versus TMB concentration when H_2_O_2_ concentration is fixed.

**S2. Calculation of AuIr loading on** **FeAu@AuIr and Fe@AuIr MNPs**

In our models (Figure S9), there is a big sphere $O_{0}$, whose radius is denoted as $r_{1}$. And there are some small balls, whose radius is denoted as $r_{2},$attached to the surface of the big sphere. We want to know how many the small balls can completely cover the surface of the big sphere. As illustrated in Figure S9c, three smaller spheres are tangential to three points on the surface of the larger sphere, and the centers of these three smaller spheres form an equilateral triangle with a side length of 2$r_{2}$. If the large sphere is fully covered by the small spheres, it is equivalent to being enclosed by a spherical shell composed of many equilateral triangles. Assuming the small spheres adhere to each other on the surface of the large sphere, they form a buckyball-like structure. One characteristic of a buckyball is that it can be composed of 12 regular pentagons and a number of regular hexagons. Let 𝑓 denote the total number of these pentagons and hexagons, $e$ denote the number of edges, and $v$ denote the number of vertices of the buckyball. Then, another geometric property of such a structure can be expressed by the following equation:

$f-e+v=2$.

Let $n_{0}$ denote the total number of vertices in Figure S9d, and $n_{1}$represent the total number of equilateral triangles. In fact, our goal is to estimate the number of vertices $n_{0}$, which corresponds to the number of small spheres. The relationship between $n_{0}$ and $n_{1}$ can be derived as follows:

$n_{1}=2n_{0}-4$.

Next, we use the area of two spheres to estimate the area of the spheroid in Figure S9d. For the two spheres, one is the minimum circumscribed sphere of the spheroid, the other is the maximum inscribed sphere of the spheroid. The radiuses of two spheres are $r_{1}+r_{2}$ and $\sqrt{r_{1}^{2}+{2r}_{1}r_{2}-\frac{1}{3}r_{2}^{2}}$, which separately refer to $O_{0}O_{1}$ and $O_{0}O_{4}$ in Figure S9e. Let $S_{2}$ and $S_{3}$ separately denote the area of the two spheres. And the area of each triangle in Figure S9d is denoted as $S_{1}$. Then, the following inequality holds:

$${S_{3}<n_{1}S_{1}<S}_{2}$$

After a series of calculation, we can estimate the $n_{0}$:

$$\frac{2\sqrt{3}\pi(r_{1}^{2}+{2r}_{1}r_{2}-\frac{1}{3}r_{2}^{2})}{3r_{2}^{2}}+2<n_{0}<\frac{2\sqrt{3}\pi{(r_{1}+r_{2})}^{2}}{3r_{2}^{2}}+2$$

For Fe@AuIr (Figure S9a), 160 nm Fe_3_O_4_ core is the big sphere, and 5 nm AuIr were the small balls, thus $r_{1}$= 160 and $r_{2}$= 5, we can estimate that the number of AuIr on Fe_3_O_4_ is at least 3946 and at most 3951.

For virus-like FeAu@AuIr (Figure S9b), the calculation needs to be carried out in two steps. In the first step, we calculate the amount of 15 nm Au NPs that can be loaded onto Fe_3_O_4_, denoted as $n_{1}$.160 nm Fe_3_O_4_ core is the big sphere, and 15 nm Au NPs are the small balls, thus $r_{1}$= 160 and $r_{2}$= 15, we can estimate $n_{2}$ is at least 490 and at most 495; In the second step, we calculate the amount of AuIr that can be loaded onto Au, denoted as $n_{2}$.15 nm Au core is the big sphere, and 5 nm AuIr NPs are the small balls, thus $r_{1}$= 15 and $r_{2}$= 5, we can estimate $n_{2}$ is at least 55 and at most 59. The amount of AuIr on FeAu@AuIr is therefore given by $n_{2}\times n_{1}$. Based on this, we estimated that the number of AuIr on FeAu surface is at least 26950 and at most 29205.

The number of AuIr loaded on FeAu MNPs is 6.8 to 7.4 times higher than that loaded on Fe_3_O_4_. This result indicates that the introduction of Au_15_ NPs as the interlayer supporter significantly increases the loading density of AuIr_5_ NPs, thereby enhancing the overall activity of the magnetic nanozyme probe and amplifying the detection signal.


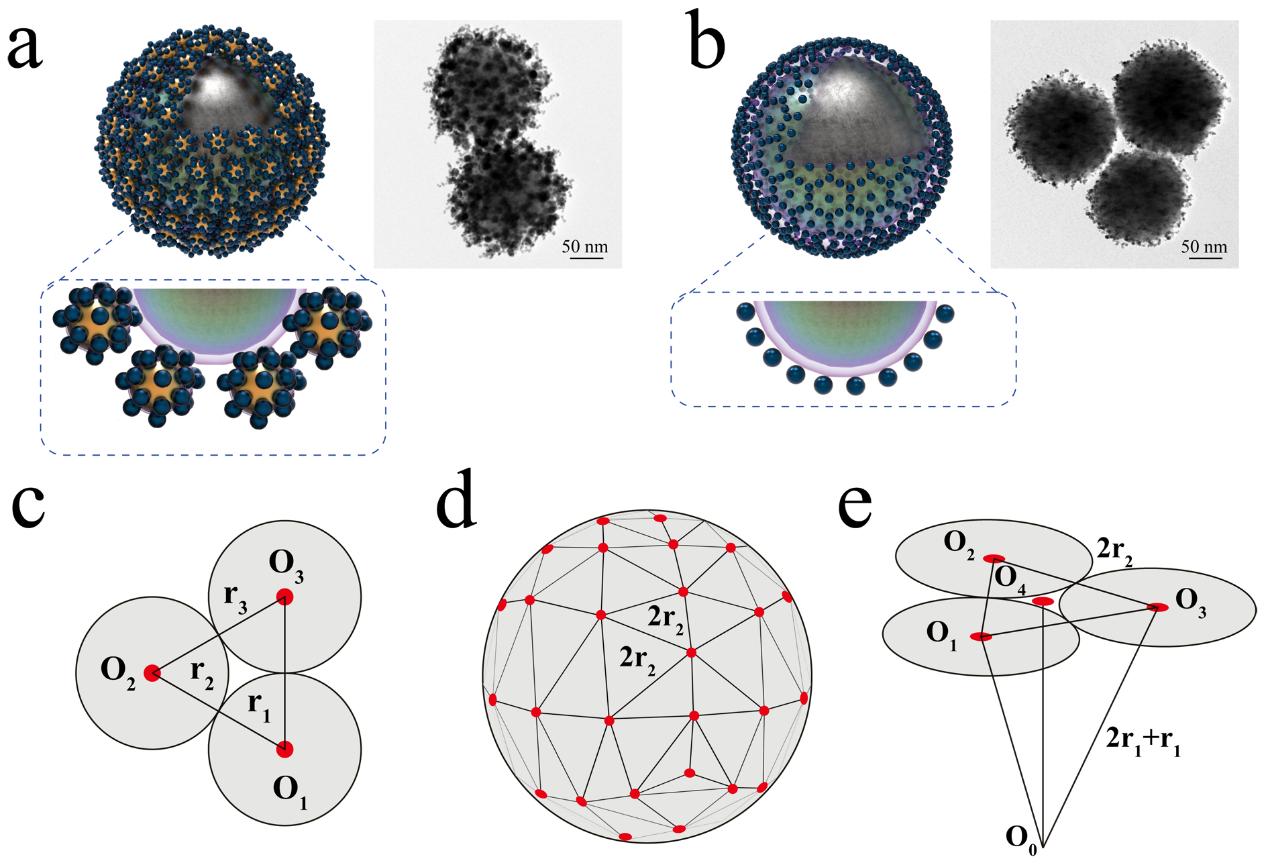


**Figure S9.** The model and typical TEM images of (a) virus-like FeAu@AuIr and (b) common Fe@AuIr nanozymes. (c-e) Distribution of small balls on the surface of the big sphere. (c) The location relationship of three small balls on the surface of the big sphere. They are tangent to three points. (d) Buckyball model composed of these smaller balls. Here, each vertex stands for a ball (center of the ball). (e) Description of the radius of two spheres used for estimating the area of the spheroid in (d). $O_{0}O_{1}$ denotes the distance of two centers of the bigger sphere and the smaller ball. The sphere with radius $O_{0}O_{1}$ is the minimum circumscribed sphere of the spheroid. $O_{0}O_{4}$ denotes the distance between the center of the big sphere and the triangle composed of three centers of the small balls. $O_{0}O_{4}\perp\triangle O_{1}O_{2}O_{3}$. The sphere with radius $O_{0}O_{4}$ is the maximum inscribed sphere of the spheroid.

**S3. Optimization of the FeAu@AuIr nanozyme size**

The size of FeAu@AuIr nanozymes was optimized in this study. As shown in Fig. S10, three kinds of FeAu@AuIr nanostructures with different Fe_3_O_4_ core (220 nm, 160 nm and 120 nm) were fabricated and tested. Fig. S10(a-c) show the TEM images of FeAu@AuIr nanostructures fabricated by 220 nm Fe_3_O_4_ core, 160 nm Fe_3_O_4_ core and 120 nm Fe_3_O_4_ core, respectively. These results indicate that the 15 nm AuNPs and 5 nm AuIr NPs could be well coated onto the 120-220 nm Fe_3_O_4_ surface through PEI-mediated electrostatic adsorption. We found that larger-sized nanozymes (Fe_220_Au@AuIr and Fe_160_Au@AuIr) exhibited superior catalytic activity compared to smaller one (Fe_120_Au@AuIr), possibly due to a higher loading of catalytic particles on the surface of larger magnetic nanozymes (Fig. S10d). In addition, the Fe_120_Au@AuIr nanozyme was limited by the weak magnetic response (30.8 emu/g), which would result in time consumption and sample wasting in the magnetic separation process (Fig. S10e). As shown in Fig. S10(f), the Fe_220_Au@AuIr and Fe_160_Au@AuIr nanozymes could be fully enriched by using a magnet in 60 s, whereas considerable amount of Fe_120_Au@AuIr MNPs could not be magnetically collected in the same time. Moreover, the Fe_220_Au@AuIr with 220 nm Fe_3_O_4_ core was found to easily block NC membrane pores on the ICA test strips, resulting in a higher background signal; however, the Fe_160_Au@AuIr nanozyme operated smoothly on the test strips, producing a higher signal-to-noise ratio.


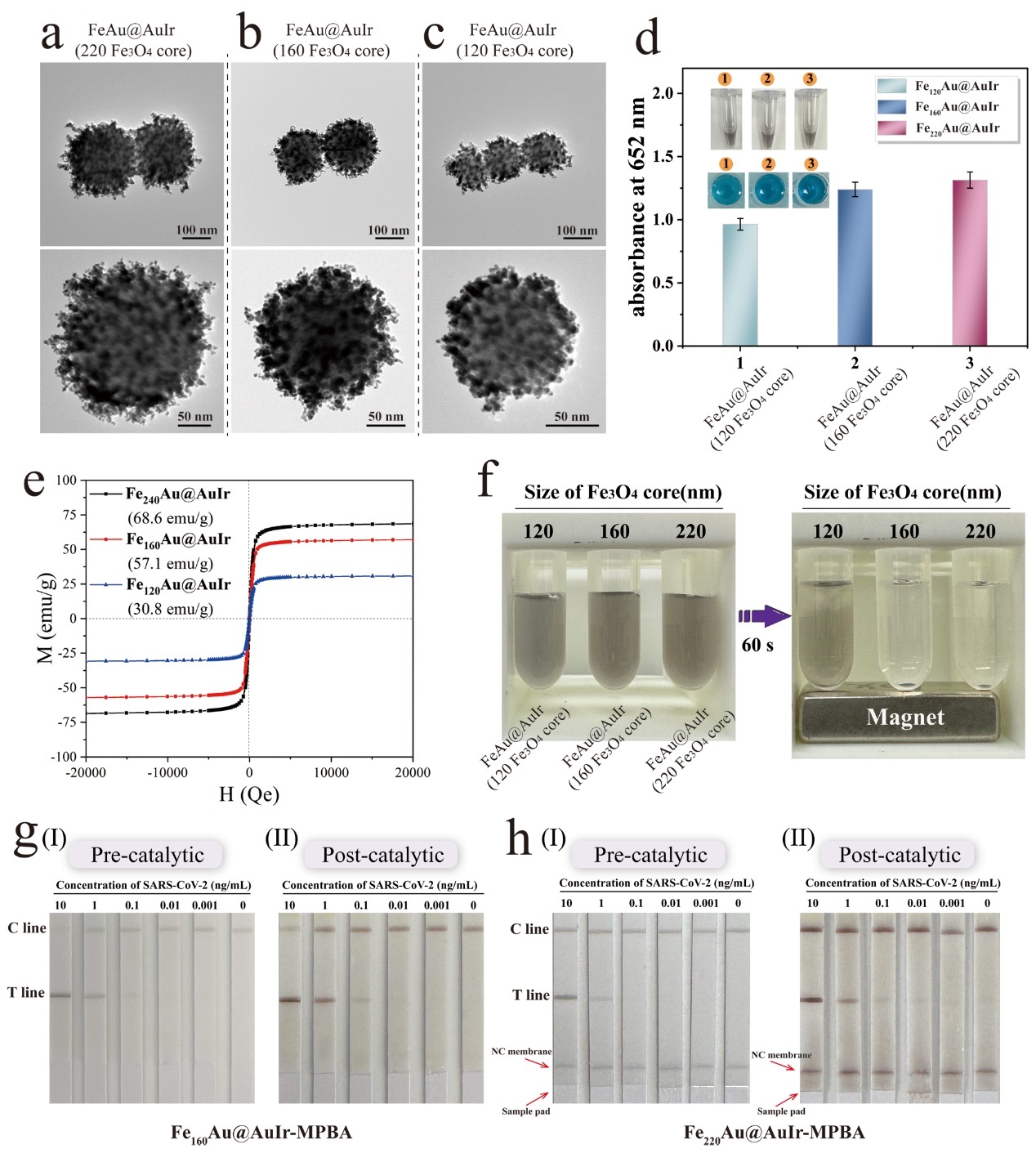


**Figure S10.** Size optimization of virus-like magnetic nanozymes by changing different Fe_3_O_4_ cores. (a-c) TEM images of biomimetic FeAu@AuIr nanozymes based on (a) 220 nm Fe_3_O_4_ MNPs, (b) 160 nm Fe_3_O_4_ MNPs, and (c) 120 nm Fe_3_O_4_ MNPs. Comparison of the (d) catalytic performance, (e) hysteresis curves, and (f) magnetic enrichment properties of the three FeAu@AuIr nanozymes. Comparison of the detection performance of (g) Fe_160_Au@AuIr and (h) Fe_220_Au@AuIr nanozymes on test strips.


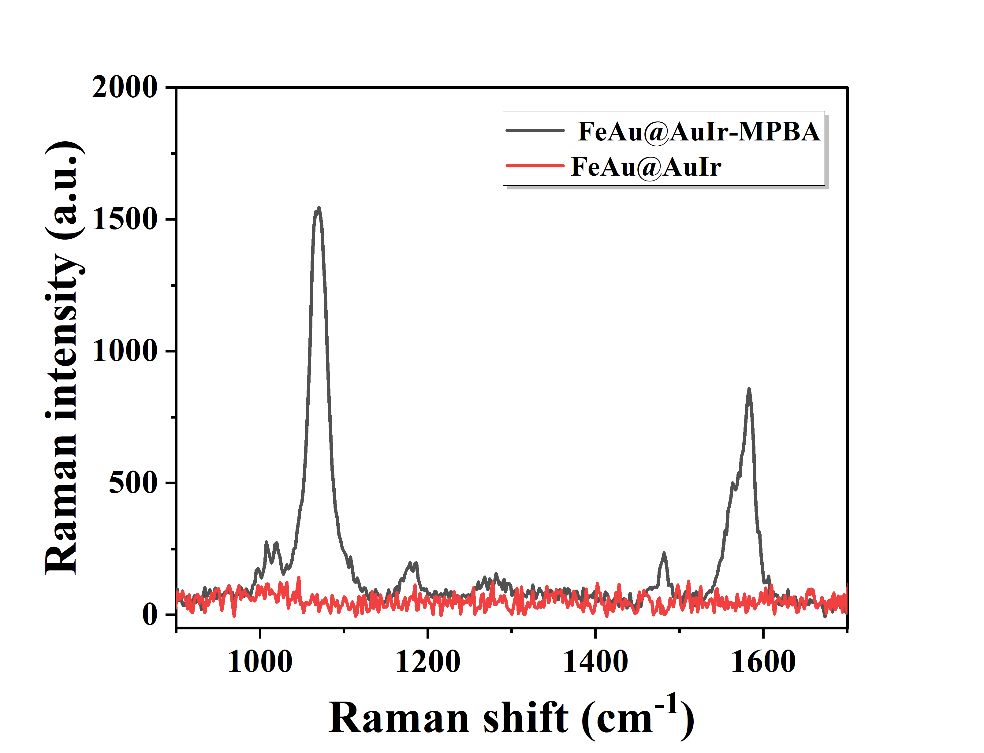


**Figure S11.** Raman spectra of FeAu@AuIr-MPBA and FeAu@AuIr.


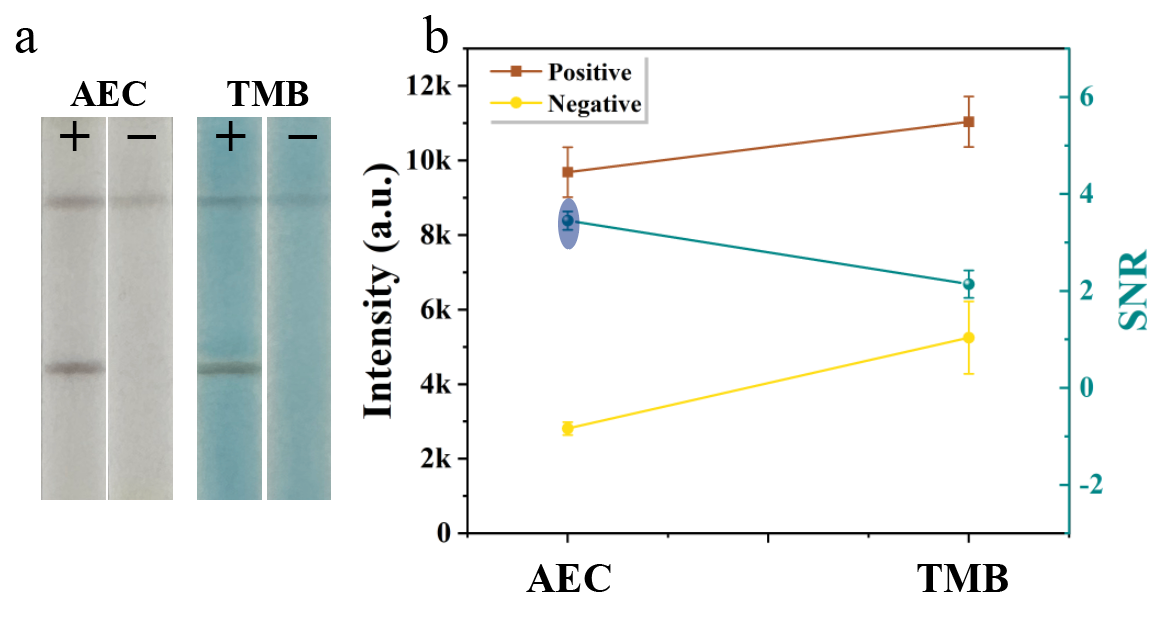


**Figure S12.** Optimization of AEC and TMB catalytic substrates. (a) Photographs of the FeAu@AuIr-based ICA for different substrates and (b) corresponding SNR.


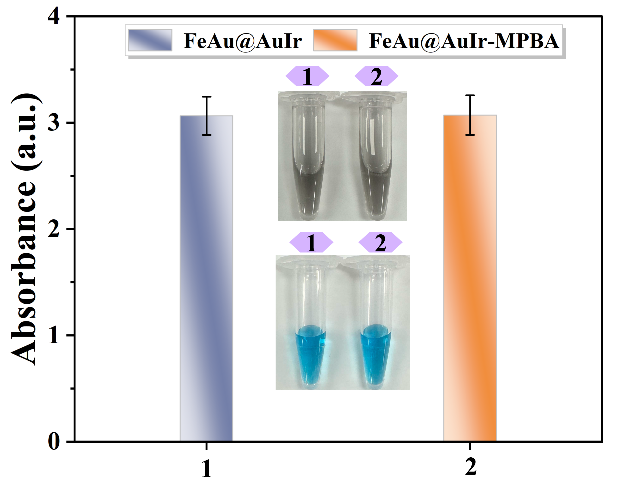


**Figure S13.** Comparison of catalytic activities of FeAu@AuIr and FeAu@AuIr-MPBA. UV–vis absorption spectra and solution color of the catalytic oxidation of TMB by identical concentrations of two nanozymes.


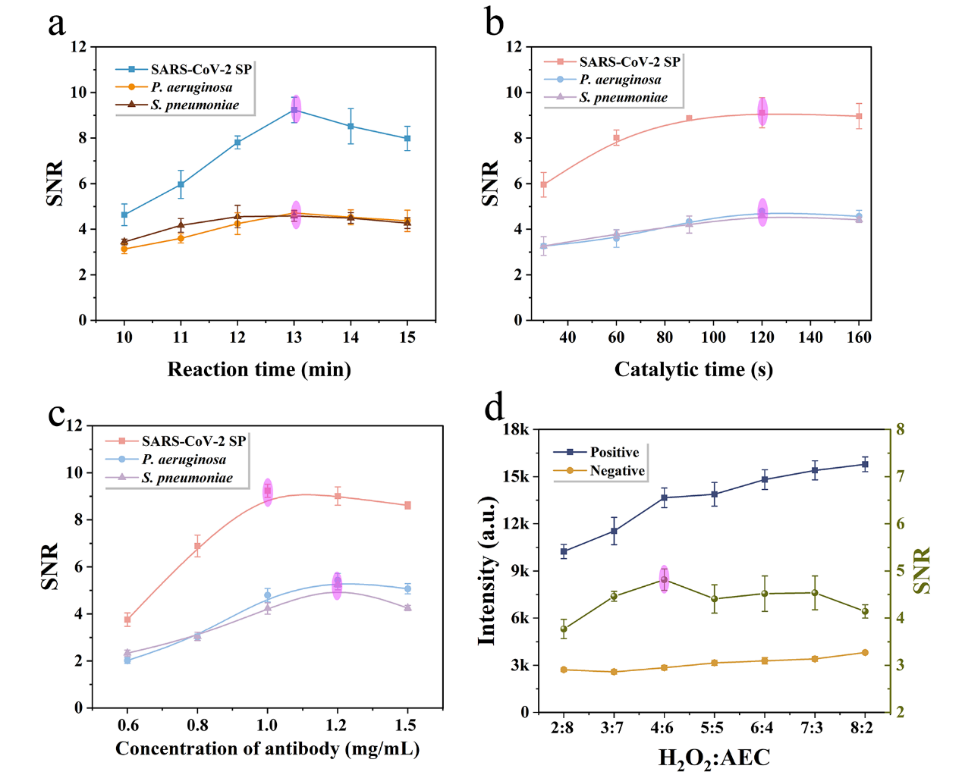


**Figure S14.** Optimization of reaction time (a), catalysis time (b), concentration of antibody on T line (c), catalytic system (d) for FeAu@AuIr-ICA.


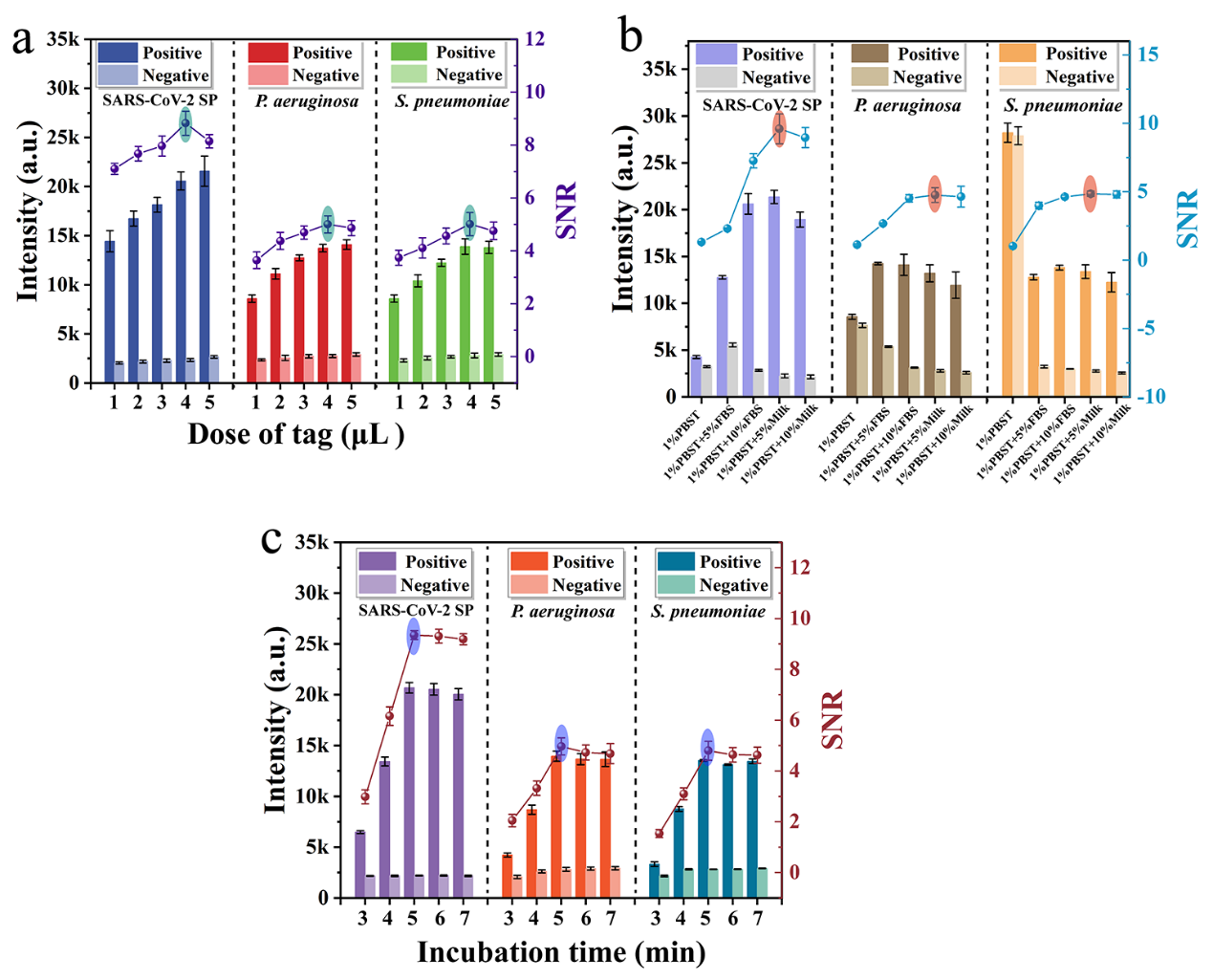


**Figure S15.** Optimization of dose of tag (a) and running buffer (b) and incubation time (c) for FeAu@AuIr-ICA.


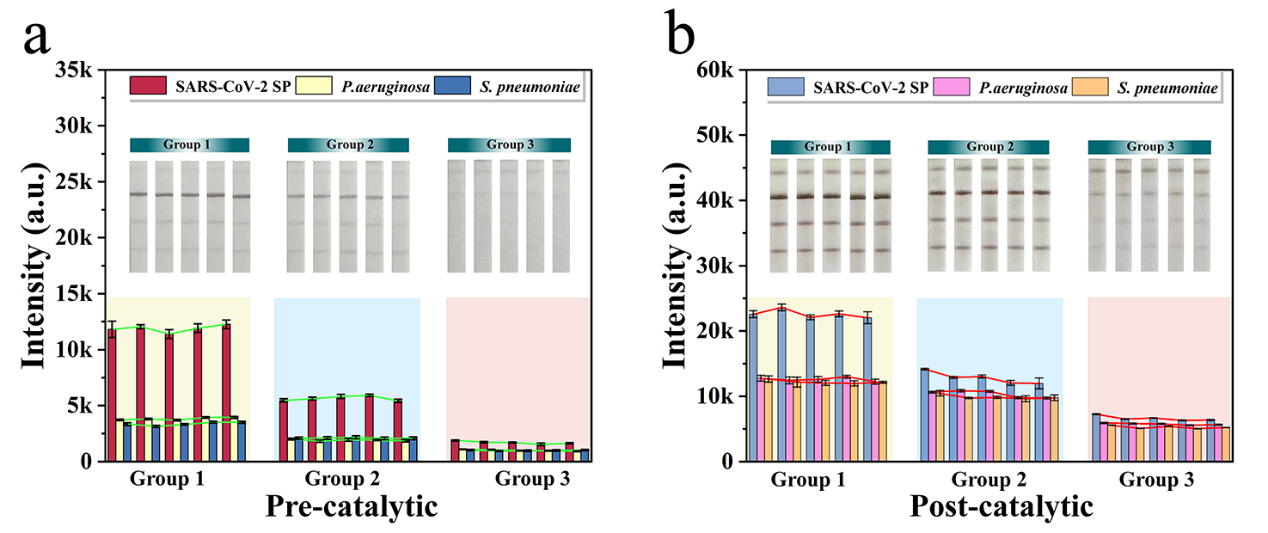


**Figure S16.** Photos and signal maps of FeAu@AuIr-ICA before (a) and after (b) catalysis at three high, medium and low concentrations.


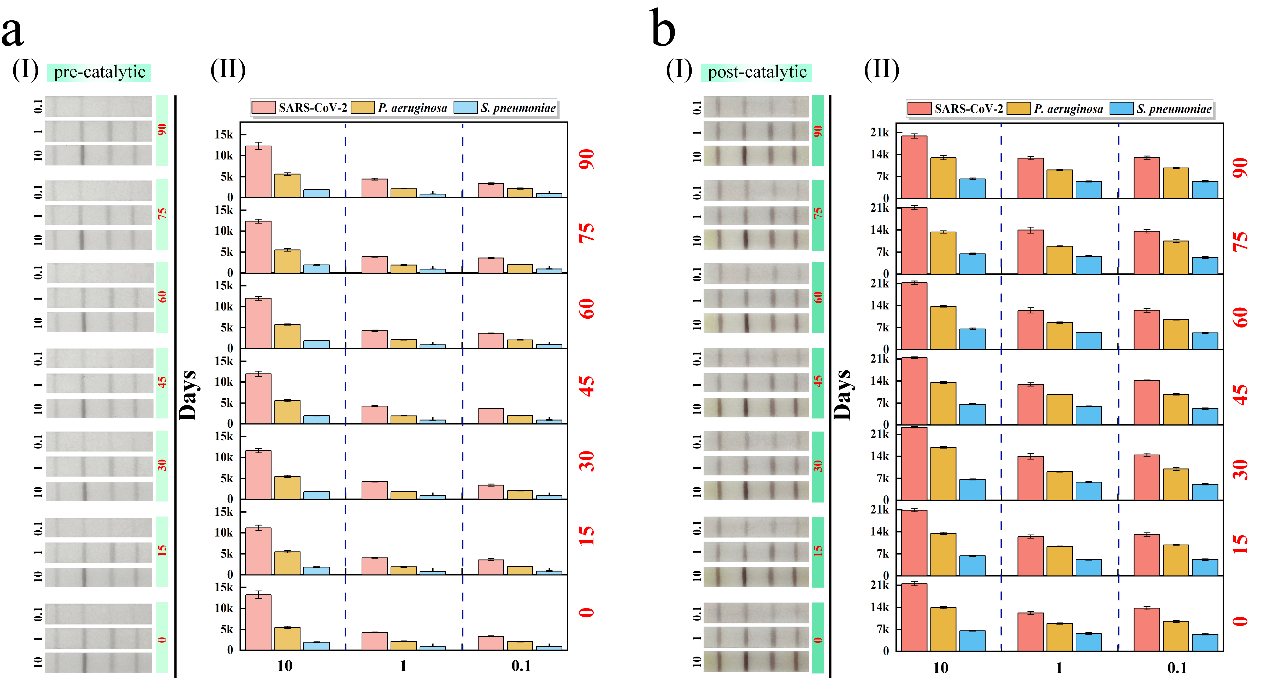


**Figure S17.** Assay stability of FeAu@AuIr-ICA stored for 90 days. Photographs (I) and corresponding colorimetric signals (c) of three T lines of the nanozyme-ICA strips before (a) and after (b) catalytic activation.

**Table S1.** Main performance of the FeAu@AuIr-based nanozyme ICA compared with other ICA-based approaches for respiratory bacteria/virus detection.

| **Signal mode** | **Pathogens** | **LODs** | **Recognition**  **molecules** | **Reference** |
| --- | --- | --- | --- | --- |
| Fluorescent | FluA, SARS-CoV-2 | 50 pfu/mL, 5 pg/mL | antibodies | Wang, 2021 [1] |
| Fluorescent | *S. pneumoniae*, *S. aureus* | 13, 20 cells/mL | antibodies | Cheng, 2021 [2] |
| Fluorescent | SARS-CoV-2 | 33 pg/mL | antibodies | Han, 2022 [3] |
| Colorimetric | SARS-CoV-2 | 10^6^ copies/mL | aptamer, antibody | Yang, 2022 [4] |
| Fluorescent | *S. pneumoniae*, FluA | 17 cells/mL;  891 copies/mL | antibodies | Cheng, 2023 [5] |
| Fluorescent | *S. pneumoniae*, *S. aureus* | 8, 13 cells/mL | MPBA, antibodies | Li, 2023 [6] |
| SERS | SARS-CoV-2 | 0.5 pg/mL | antibodies | Lin, 2023 [7] |
| Colorimetric | SARS-CoV-2, FluA | 1.3, 0.84 pg/mL | antibodies | Wang, 2024 [8] |
| Colorimetric | FluA | 8 pg/mL | antibodies | Liang, 2025 [9] |
| Fluorescent | SARS-CoV-2, MPXV | 5.6, 2.9 pg/mL | WGA, antibodies | Wu, 2025 [10] |
| Colorimetric | *P. aeruginosa*, *S. pneumoniae*, SARS-CoV-2 | 17, 21 cells/mL, 1.2 pg/mL | MPBA, antibodies | This work |

**References**

1. Wang C, Yang X, Zheng S, Cheng X, Xiao R, Li Q, Wang W, Liu X, Wang S. Development of an ultrasensitive fluorescent immunochromatographic assay based on multilayer quantum dot nanobead for simultaneous detection of SARS-CoV-2 antigen and influenza A virus. ***Sens Actuators B Chem.*** 2021, 345, 130372.
2. Cheng X, Zheng S, Wang W, Han H, Yang X, Shen W, Wang C, Wang S. Synthesis of two-dimensional graphene oxide-fluorescent nanoprobe for ultrasensitive and multiplex immunochromatographic detection of respiratory bacteria. ***Chemical Engineering Journal*** 2021, 426, 131836.
3. Han H, Wang C, Yang X, Zheng S, Cheng X, Liu Z, Zhao B, Xiao R. Rapid field determination of SARS-CoV-2 by a colorimetric and fluorescent dual-functional lateral flow immunoassay biosensor. ***Sens Actuators B Chem.*** 2022, 351, 130897.
4. Yang LF, Kacherovsky N, Panpradist N, Wan R, Liang J, Zhang B, Salipante SJ, Lutz BR, Pun SH. Aptamer Sandwich Lateral Flow Assay (AptaFlow) for Antibody-Free SARS-CoV-2 Detection. ***Anal Chem.*** 2022, 94(20), 7278-7285.
5. Cheng X, Yang X, Tu Z, Rong Z, Wang C, Wang S. Graphene oxide-based colorimetric/fluorescence dual-mode immunochromatography assay for simultaneous ultrasensitive detection of respiratory virus and bacteria in complex samples. ***J Hazard Mater.*** 2023, 459, 132192.
6. Li J, Chen J, Dai Y, Liu Z, Zhao J, Liu S, Xiao R. Magnetic SERS Strip Based on 4-mercaptophenylboronic Acid-Modified Fe_3_O_4_@Au for Active Capture and Simultaneous Detection of Respiratory Bacteria. ***Biosensors (Basel)***. 2023, 13(2), 210.
7. Lin C, Liu Z, Fang F, Zhao S, Li Y, Xu M, Peng Y, Chen H, Yuan F, Zhang W, Zhang X, Teng Z, Xiao R, Yang Y. Next-Generation Rapid and Ultrasensitive Lateral Flow Immunoassay for Detection of SARS-CoV-2 Variants. ***ACS Sens.*** 2023, 8(10), 3733-3743.
8. Wang C, Shen W, Li Z, Xia X, Li J, Xu C, Zheng S, Gu B. 3D Film-Like Nanozyme with a Synergistic Amplification Effect for the Ultrasensitive Immunochromatographic Detection of Respiratory Viruses. ***ACS Nano.*** 2024, 18(37), 25865-25879.
9. Liang J, Liu X, Liu Z, Xu X, Sun Y, Chen Y, Xiao R, Wang Y. Nanozymes featuring a mesoporous silica shell for rapid enrichment and ultrasensitive lateral flow immunoassay of influenza A. ***Anal Chim Acta.*** 2025, 1335, 343474.
10. Wu T, Liu Y, Zhou S, Li J, Sun G, Gu B, Wang C. Wheat Germ Agglutinin-Modified "Three-in-One" Multifunctional Probe Driven Broad-Spectrum and Flexible Immunochromatographic Diagnosis of viruses With High Sensitivity. ***Small.*** 2025, 21(10), e2406053.

**Table S2.** The reproducibility of the FeAu@AuIr-MPBA-ICA for SARS-CoV-2 SP, *P. aeruginosa*, and *S. pneumoniae* in spiked sample solutions before catalysis (n=3).

| **samples** | **pathogen** | **Add concentration** | **Detected concentration** | **Recovey (%)** | **RSD (%)** |
| --- | --- | --- | --- | --- | --- |
| **Throat swab** | **SARS-CoV-2 SP** | 10 ng/mL | 10.643 ng/mL | 106.43 | 11.83 |
|  |  | 1 ng/mL | 1.031 ng/mL | 103.13 | 5.92 |
|  |  | 0. 1 ng/mL | 0.086 ng/mL | 86.19 | 7.40 |
|  |  | 0.01 ng/mL | 0.011 ng/mL | 115.60 | 10.67 |
|  | ***P. aeruginos*a** | 10^5^ cells/mL | 101276 cells/mL | 101.28 | 8.50 |
|  |  | 10^4^ cells/mL | 9348 cells/mL | 93.48 | 7.93 |
|  |  | 10^3^ cells/mL | 1170 cells/mL | 11.70 | 5.84 |
|  |  | 10^2^ cells/mL |  |  |  |
|  | ***S. pneumoniae*** | 10^5^ cells/mL | 99524 cells/mL | 99.52 | 7.41 |
|  |  | 10^4^ cells/mL | 11594 cells/mL | 115.94 | 8.73 |
|  |  | 10^3^ cells/mL | 1109 cells/mL | 110.90 | 6.86 |
|  |  | 10^2^ cells/mL |  |  |  |

**Table S3.** The reproducibility of the FeAu@AuIr-ICA for SARS-CoV-2 SP, *P. aeruginosa*, and *S. pneumoniae* in spiked sample solutions after catalysis (n=3).

| **samples** | **pathogen** | **Add concentration** | **Detected concentration** | **Recovery (%)** | **RSD (%)** |
| --- | --- | --- | --- | --- | --- |
| **Throat swab** | **SARS-CoV-2 SP** | 10 ng/mL | 10.798 ng/mL | 107.98 | 11.13 |
|  |  | 1 ng/mL | 0.884 ng/mL | 88.44 | 8.88 |
|  |  | 0. 1 ng/mL | 0.0873 ng/mL | 87.30 | 6.66 |
|  |  | 0.01 ng/mL | 0.011 ng/mL | 110.09 | 11.30 |
|  | ***P. aeruginos*a** | 10^5^ cells/mL | 87080 cells/mL | 87.08 | 12.67 |
|  |  | 10^4^ cells/mL | 109.62 cells/mL | 109.62 | 12.69 |
|  |  | 10^3^ cells/mL | 1110 cells/mL | 111.00 | 9.74 |
|  |  | 10^2^ cells/mL | 108 cells/mL | 108.00 | 4.72 |
|  | ***S. pneumoniae*** | 10^5^ cells/mL | 106306 cells/mL | 106.31 | 9.69 |
|  |  | 10^4^ cells/mL | 10113 cells/mL | 101.13 | 9.54 |
|  |  | 10^3^ cells/mL | 903 cells/mL | 90.30 | 4.53 |
|  |  | 10^2^ cells/mL | 108 cells/mL | 108.00 | 4.87 |

**Table S4.** Quantification of 59 SARS-CoV-2 positive throat swab samples determined by the FeAu@AuIr-ICA (catalytic mode) and ELISA.

| **Clinical**  **samples** | **FeAu@AuIr-ICA(catalytic mode)** | | **ELISA** |
| --- | --- | --- | --- |
|  | **Classification** | **Quantification** | **Quantification** |
| 1 | SARS-CoV-2 | 8.37 ng/mL | >5 ng/mL |
| 2 | SARS-CoV-2 | 1.61 ng/mL | 1.12 ng/mL |
| 3 | SARS-CoV-2 | 2.13 ng/mL | 2.59 ng/mL |
| 4 | SARS-CoV-2 | 0.55 ng/mL | 0.68 ng/mL |
| 5 | SARS-CoV-2 | 3.03 ng/mL | 3.37 ng/mL |
| 6 | SARS-CoV-2 | 1.28 ng/mL | 0.92 ng/mL |
| 7 | SARS-CoV-2 | 0.97 ng/mL | 0.48 ng/mL |
| 8 | SARS-CoV-2 | 1.04 ng/mL | 1.62 ng/mL |
| 9 | SARS-CoV-2 | 5.06 ng/mL | 4.85 ng/mL |
| 10 | SARS-CoV-2 | 8.99 ng/mL | >5 ng/mL |
| 11 | SARS-CoV-2 | 4.49 ng/mL | 4.28 ng/mL |
| 12 | SARS-CoV-2 | 3.00 ng/mL | 2.75 ng/mL |
| 13 | SARS-CoV-2 | 4.31 ng/mL | 3.74 ng/mL |
| 14 | SARS-CoV-2 | 4.45 ng/mL | 4.92 ng/mL |
| 15 | SARS-CoV-2 | 2.05 ng/mL | 1.92 ng/mL |
| 16 | SARS-CoV-2 | 3.57 ng/mL | 3.91 ng/mL |
| 17 | SARS-CoV-2 | 0.54 ng/mL | 0.23 ng/mL |
| 18 | SARS-CoV-2 | 0.92 ng/mL | 1.06 ng/mL |
| 19 | SARS-CoV-2 | 3.98 ng/mL | 3.61 ng/mL |
| 20 | SARS-CoV-2 | 5.00 ng/mL | 4.88 ng/mL |
| 21 | SARS-CoV-2 | 0.11 ng/mL | 0.18 ng/mL |
| 22 | SARS-CoV-2 | 1.33 ng/mL | 0.97 ng/mL |
| 23 | SARS-CoV-2 | 3.76 ng/mL | 4.07 ng/mL |
| 24 | SARS-CoV-2 | 1.27 ng/mL | 1.39 ng/mL |
| 25 | SARS-CoV-2 | 4.84 ng/mL | 4.39 ng/mL |
| 26 | SARS-CoV-2 | 1.37 ng/mL | 1.61 ng/mL |
| 27 | SARS-CoV-2 | 4.58 ng/mL | 4.30 ng/mL |
| 28 | SARS-CoV-2 | 0.38 ng/mL | 0.32 ng/mL |
| 29 | SARS-CoV-2 | 0.23 ng/mL | 0.19 ng/mL |
| 30 | SARS-CoV-2 | 7.64 ng/mL | >5 ng/mL |
| 31 | SARS-CoV-2 | 2.31 ng/mL | 2.39 ng/mL |
| 32 | SARS-CoV-2 | 3.36 ng/mL | 3.20 ng/mL |
| 33 | SARS-CoV-2 | 0.10 ng/mL | 0.16 ng/mL |
| 34 | SARS-CoV-2 | 1.45 ng/mL | 1.09 ng/mL |
| 35 | SARS-CoV-2 | 0.97 ng/mL | 1.26 ng/mL |
| 36 | SARS-CoV-2 | 1.00 ng/mL | 0.72 ng/mL |
| 37 | SARS-CoV-2 | 4.08 ng/mL | 4.59 ng/mL |
| 38 | SARS-CoV-2 | 2.43 ng/mL | 2.21 ng/mL |
| 39 | SARS-CoV-2 | 4.13 ng/mL | 3.37 ng/mL |
| 40 | SARS-CoV-2 | 0.65 ng/mL | 0.82 ng/mL |
| 41 | SARS-CoV-2 | 5.03 ng/mL | 4.83 ng/mL |
| 42 | SARS-CoV-2 | 1.04 ng/mL | 1.25 ng/mL |
| 43 | SARS-CoV-2 | 1.56 ng/mL | 1.98 ng/mL |
| 44 | SARS-CoV-2 | 0.16 ng/mL | 0.11 ng/mL |
| 45 | SARS-CoV-2 | 3.20 ng/mL | 3.48 ng/mL |
| 46 | SARS-CoV-2 | 0.25 ng/mL | 0.14 ng/mL |
| 47 | SARS-CoV-2 | 2.43 ng/mL | 2.28 ng/mL |
| 48 | SARS-CoV-2 | 3.61 ng/mL | 4.04 ng/mL |
| 49 | SARS-CoV-2 | 0.57 ng/mL | 0.46 ng/mL |
| 50 | SARS-CoV-2 | 0.16 ng/mL | 0.18 ng/mL |
| 51 | SARS-CoV-2 | 4.26 ng/mL | 4.35 ng/mL |
| 52 | SARS-CoV-2 | 2.45 ng/mL | 2.74 ng/mL |
| 53 | SARS-CoV-2 | 0.20 ng/mL | 0.21 ng/mL |
| 54 | SARS-CoV-2 | 1.41 ng/mL | 1.36 ng/mL |
| 55 | SARS-CoV-2 | 5.45 ng/mL | 4.75 ng/mL |
| 56 | SARS-CoV-2 | 3.94 ng/mL | 3.57 ng/mL |
| 57 | SARS-CoV-2 | 0.12 ng/mL | 0.14 ng/mL |
| 58 | SARS-CoV-2 | 1.42 ng/mL | 1.05 ng/mL |
| 59 | SARS-CoV-2 | 0.29 ng/mL | 0.42 ng/mL |

**Table S5.** Quantification of 59 *P. aeruginosa* positive bronchoalveolar lavage fluid samples and 52 *S. pneumoniae* positive bronchoalveolar lavage fluid samples determined by the FeAu@AuIr- ICA (catalytic mode) and qPCR.

| **Clinical**  **samples** | **FeAu@AuIr-ICA(catalytic mode)** | | **qPCR** |
| --- | --- | --- | --- |
|  | **Classification** | **Quantification** | **Quantification** |
| 1 | *P. aeruginosa* | 677 cells/mL | 948 cells/mL |
| 2 | *P. aeruginosa* | 1723 cells/mL | 2033 cells/mL |
| 3 | *P. aeruginosa* | 79753 cells/mL | 76202 cells/mL |
| 4 | *P. aeruginosa* | 17387 cells/mL | 20028 cells/mL |
| 5 | *P. aeruginosa* | 1960 cells/mL | 2668 cells/mL |
| 6 | *P. aeruginosa* | 448 cells/mL | 612 cells/mL |
| 7 | *P. aeruginosa* | 99 cells/mL | 55 cells/mL |
| 8 | *P. aeruginosa* | 75966 cells/mL | 92728 cells/mL |
| 9 | *P. aeruginosa* | 14758 cells/mL | 19727 cells/mL |
| 10 | *P. aeruginosa* | 71653 cells/mL | 94852 cells/mL |
| 11 | *P. aeruginosa* | 353 cells/mL | 204 cells/mL |
| 12 | *P. aeruginosa* | 16605 cells/mL | 13322 cells/mL |
| 13 | *P. aeruginosa* | 547 cells/mL | 821 cells/mL |
| 14 | *P. aeruginosa* | 89447 cells/mL | 97025 cells/mL |
| 15 | *P. aeruginosa* | 55028 cells/mL | 201599 cells/mL |
| 16 | *P. aeruginosa* | 71416 cells/mL | 83427 cells/mL |
| 17 | *P. aeruginosa* | 71416 cells/mL | 83427 cells/mL |
| 18 | *P. aeruginosa* | 8857 cells/mL | 11369 cells/mL |
| 19 | *P. aeruginosa* | 332 cells/mL | 180 cells/mL |
| 20 | *P. aeruginosa* | 4809 cells/mL | 5939 cells/mL |
| 21 | *P. aeruginosa* | 459 cells/mL | 506 cells/mL |
| 22 | *P. aeruginosa* | 228 cells/mL | 256 cells/mL |
| 23 | *P. aeruginosa* | 2945 cells/mL | 2608 cells/mL |
| 24 | *P. aeruginosa* | 65552 cells/mL | 58067 cells/mL |
| 25 | *P. aeruginosa* | 29434 cells/mL | 25888 cells/mL |
| 26 | *P. aeruginosa* | 12354 cells/mL | 8280 cells/mL |
| 27 | *P. aeruginosa* | 3729 cells/mL | 5591 cells/mL |
| 28 | *P. aeruginosa* | 6990 cells/mL | 4808 cells/mL |
| 29 | *P. aeruginosa* | 1770 cells/mL | 1323 cells/mL |
| 30 | *P. aeruginosa* | 556 cells/mL | 970 cells/mL |
| 31 | *P. aeruginosa* | 8677 cells/mL | 12926 cells/mL |
| 32 | *P. aeruginosa* | 242 cells/mL | 148 cells/mL |
| 33 | *P. aeruginosa* | 72522 cells/mL | 94852 cells/mL |
| 34 | *P. aeruginosa* | 1420 cells/mL | 744 cells/mL |
| 35 | *P. aeruginosa* | 471 cells/mL | 329 cells/mL |
| 36 | *P. aeruginosa* | 46035 cells/mL | 42932 cells/mL |
| 37 | *P. aeruginosa* | 28934 cells/mL | 21274 cells/mL |
| 38 | *P. aeruginosa* | 1176 cells/mL | 1478 cells/mL |
| 39 | *P. aeruginosa* | 15334 cells/mL | 25694 cells/mL |
| 40 | *P. aeruginosa* | 3090 cells/mL | 1857 cells/mL |
| 41 | *P. aeruginosa* | 141 cells/mL | 92 cells/mL |
| 42 | *P. aeruginosa* | 453 cells/mL | 334 cells/mL |
| 43 | *P. aeruginosa* | 279 cells/mL | 342 cells/mL |
| 44 | *P. aeruginosa* | 57088 cells/mL | 82177 cells/mL |
| 45 | *P. aeruginosa* | 2640 cells/mL | 1514 cells/mL |
| 46 | *P. aeruginosa* | 619 cells/mL | 374 cells/mL |
| 47 | *P. aeruginosa* | 70652 cells/mL | 95571 cells/mL |
| 48 | *P. aeruginosa* | 55139 cells/mL | 48811 cells/mL |
| 49 | *P. aeruginosa* | 5559 cells/mL | 4844 cells/mL |
| 50 | *P. aeruginosa* | 11181 cells/mL | 20332 cells/mL |
| 51 | *P. aeruginosa* | 66705 cells/mL | 64055 cells/mL |
| 52 | *P. aeruginosa* | 41735 cells/mL | 76202 cells/mL |
| 53 | *P. aeruginosa* | 1860 cells/mL | 1198 cells/mL |
| 54 | *P. aeruginosa* | 1176 cells/mL | 791 cells/mL |
| 55 | *P. aeruginosa* | 38316 cells/mL | 50689 cells/mL |
| 56 | *P. aeruginosa* | 17830 cells/mL | 24371 cells/mL |
| 57 | *P. aeruginosa* | 610 cells/mL | 791cells/mL |
| 58 | *P. aeruginosa* | 3769 cells/mL | 2192 cells/mL |
| 59 | *P. aeruginosa* | 21285 cells/mL | 11199 cells/mL |
| 60 | *S. pneumoniae* | 6778 cells/mL | 7913 cells/mL |
| 61 | *S. pneumoniae* | 1731 cells/mL | 2400 cells/mL |
| 62 | *S. pneumoniae* | 20756 cells/mL | 22260 cells/mL |
| 63 | *S. pneumoniae* | 209 cells/mL | 177 cells/mL |
| 64 | *S. pneumoniae* | 129 cells/mL | 189 cells/mL |
| 65 | *S. pneumoniae* | 10288 cells/mL | 12829 cells/mL |
| 66 | *S. pneumoniae* | 111 cells/mL | 94 cells/mL |
| 67 | *S. pneumoniae* | 18640 cells/mL | 13423 cells/mL |
| 68 | *S. pneumoniae* | 14725 cells/mL | 10229 cells/mL |
| 69 | *S. pneumoniae* | 729 cells/mL | 607 cells/mL |
| 70 | *S. pneumoniae* | 73 cells/mL | 63 cells/mL |
| 71 | *S. pneumoniae* | 123 cells/mL | 107 cells/mL |
| 72 | *S. pneumoniae* | 12 cells/mL | 26 cells/mL |
| 73 | *S. pneumoniae* | 18790 cells/mL | 15147 cells/mL |
| 74 | *S. pneumoniae* | 2043 cells/mL | 1928 cells/mL |
| 75 | *S. pneumoniae* | 551 cells/mL | 859 cells/mL |
| 76 | *S. pneumoniae* | 665 cells/mL | 690 cells/mL |
| 77 | *S. pneumoniae* | 2975 cells/mL | 2259 cells/mL |
| 78 | *S. pneumoniae* | 330 cells/mL | 249 cells/mL |
| 79 | *S. pneumoniae* | 620 cells/mL | 404 cells/mL |
| 80 | *S. pneumoniae* | 286 cells/mL | 277 cells/mL |
| 81 | *S. pneumoniae* | 90 cells/mL | 120 cells/mL |
| 82 | *S. pneumoniae* | 1180 cells/mL | 2209 cells/mL |
| 83 | *S. pneumoniae* | 2158 cells/mL | 1189 cells/mL |
| 84 | *S. pneumoniae* | 7237 cells/mL | 9485 cells/mL |
| 85 | *S. pneumoniae* | 3850 cells/mL | 2921 cells/mL |
| 86 | *S. pneumoniae* | 9281 cells/mL | 11806 cells/mL |
| 87 | *S. pneumoniae* | 21701 cells/mL | 27709 cells/mL |
| 88 | *S. pneumoniae* | 5453 cells/mL | 3608 cells/mL |
| 89 | *S. pneumoniae* | 102 cells/mL | 70 cells/mL |
| 90 | *S. pneumoniae* | 44732 cells/mL | 56340 cells/mL |
| 91 | *S. pneumoniae* | 61 cells/mL | 34 cells/mL |
| 92 | *S. pneumoniae* | 39791 cells/mL | 33718 cells/mL |
| 93 | *S. pneumoniae* | 246 cells/mL | 150 cells/mL |
| 94 | *S. pneumoniae* | 4737 cells/mL | 5763 cells/mL |
| 95 | *S. pneumoniae* | 2953 cells/mL | 1189 cells/mL |
| 96 | *S. pneumoniae* | 19149 cells/mL | 26884 cells/mL |
| 97 | *S. pneumoniae* | 729 cells/mL | 680 cells/mL |
| 98 | *S. pneumoniae* | 41150 cells/mL | 63573 cells/mL |
| 99 | *S. pneumoniae* | 2995 cells/mL | 2608 cells/mL |
| 100 | *S. pneumoniae* | 31878 cells/mL | 29657 cells/mL |
| 101 | *S. pneumoniae* | 285 cells/mL | 227 cells/mL |
| 102 | *S. pneumoniae* | 137 cells/mL | 75 cells/mL |
| 103 | *S. pneumoniae* | 6060 cells/mL | 5939 cells/mL |
| 104 | *S. pneumoniae* | 84 cells/mL | 62 cells/mL |
| 105 | *S. pneumoniae* | 66415 cells/mL | 78538 cells/mL |
| 106 | *S. pneumoniae* | 21206 cells/mL | 31982 cells/mL |
| 107 | *S. pneumoniae* | 67 cells/mL | 76 cells/mL |
| 108 | *S. pneumoniae* | 469 cells/mL | 337 cells/mL |
| 109 | *S. pneumoniae* | 2791 cells/mL | 2364 cells/mL |
| 110 | *S. pneumoniae* | 48155 cells/mL | 48444 cells/mL |
| 111 | *S. pneumoniae* | 223 cells/mL | 76 cells/mL |
